# Identifying genetic diversity of O antigens in *Aeromonas hydrophila* for molecular serotype detection

**DOI:** 10.1371/journal.pone.0203445

**Published:** 2018-09-05

**Authors:** Hengchun Cao, Min Wang, Qian Wang, Tingting Xu, Yuhui Du, Huiying Li, Chengqian Qian, Zhiqiu Yin, Lu Wang, Yi Wei, Pan Wu, Xi Guo, Bin Yang, Bin Liu

**Affiliations:** 1 Key Laboratory of Molecular Microbiology and Technology, Ministry of Education, Tianjin Economic-Technological Development Area, Tianjin, China; 2 Tianjin Key Laboratory of Microbial Functional Genomics, Tianjin Economic-Technological Development Area, Tianjin, China; 3 TEDA Institute of Biological Sciences and Biotechnology, Nankai University, Tianjin Economic-Technological Development Area, Tianjin, China; The Pennsylvania State University, UNITED STATES

## Abstract

*Aeromonas hydrophila* is a globally occurring, potentially virulent, gram-negative opportunistic pathogen that is known to cause water and food-borne diseases around the world. In this study, we use whole genome sequencing and *in silico* analyses to identify 14 putative O antigen gene clusters (OGCs) located downstream of the housekeeping genes *acrB* and/or *oprM*. We have also identified 7 novel OGCs by analyzing 15 publicly available genomes of different *A*. *hydrophila* strains. From the 14 OGCs identified initially, we have deduced that O antigen processing genes involved in the *wzx*/*wzy* pathway and the ABC transporter (*wzm*/*wzt*) pathway exhibit high molecular diversity among different *A*. *hydrophila* strains. Using these genes, we have developed a multiplexed Luminex-based array system that can identify up to 14 *A*. *hydrophila* strains. By combining our other results and including the sequences of processing genes from 13 other OGCs (7 OGCs identified from publicly available genome sequences and 6 OGCs that were previously published), we also have the data to create an array system that can identify 25 different *A*. *hydrophila* serotypes. Although clinical detection, epidemiological surveillance, and tracing of pathogenic bacteria are typically done using serotyping methods that rely on identifying bacterial surface O antigens through agglutination reactions with antisera, molecular methods such as the one we have developed may be quicker and more cost effective. Our assay shows high specificity, reproducibility, and sensitivity, being able to classify *A*. *hydrophila* strains using just 0.1 ng of genomic DNA. In conclusion, our findings indicate that a molecular serotyping system for *A*. *hydrophila* could be developed based on specific genes, providing an important molecular tool for the identification of *A*. *hydrophila* serotypes.

## Introduction

*Aeromonas hydrophila* is a rod-shaped, motile, gram-negative bacterium, which is widely distributed in aquatic environments such as wastewater, sewage, and drinking water, as well as in food [1,2]. It is the most common opportunistic pathogen found in poikilotherms such as fish, and is also an important human pathogen [3,4]. Different strains of *A*. *hydrophila* are known to cause not only skin infections and gastroenteritis, but also systemic problems such as meningitis, peritonitis, necrotizing fasciitis, and hemolytic uremic syndrome[5]. Immunocompromised patients with hepatic diseases, trauma, or cancer, are thought be at high risk of developing fatal *A*. *hydrophila* infections and sepsis [6,7]. The pathogeneses of *A*. *hydrophila* infections are complex, multifactorial, and dependent on several virulence factors such as O antigens, capsules, lipopolysaccharides (LPS), S-layers, exotoxins, iron-binding systems, and secretion systems [8–13].

Of these virulence factors, bacterial polysaccharides such as capsular polysaccharides (K antigens) and O polysaccharides (O antigens) that are present on bacterial cell surfaces are extremely important for host—pathogen interactions as they play crucial roles in adhesion, cell recognition, and biofilm formation, [14–16]. The O antigen gene cluster (OGC) is normally located between the *galF* and *gnd* genes in *Salmonella* spp., *Escherichia coli*, and *Shigella* spp. [17]. The genes involved in O antigen synthesis are classified into three main groups: 1) nucleotide sugar synthesis genes; 2) genes for glycosyltransferases (GTs), which add sugars to a growing repeat unit; and 3) processing genes, including those encoding the flippase and polymerase proteins [17–19]. In addition to these three gene classes, the integrated inner membrane protein Wzz is involved in regulating the chain length of the O antigen, and is an essential virulence factor in many pathogens. The fully-synthesized O antigen is then ligated to the lipid A/core to form the complete LPS by WaaL, which is encoded by the *waaL* gene, also located in the core gene cluster [20,21].

The structural diversities of bacterial surface polysaccharides were first detected in the form of antigenic diversities. The antigenic diversities of surface polysaccharides in bacteria are widely used in serotyping to identify and detect bacterial strains; this is often invaluable for epidemiological investigations. For example, many O antigen-based serotypes are associated with specific disease syndromes, such as meningitis, systemic infections, or diarrhea [22]. However, conventional serotyping processes involving agglutination using antisera are laborious, time consuming, and impractical when analyzing large numbers of specimens. Furthermore, serological cross-reactions can create ambiguities in serotype identifications, and rough strains that do not produce surface antigens are unidentifiable using this method [23,24]. Although no new method has completely replaced bacterial strain identification by conventional serotyping [25], DNA-based typing methods involving polysaccharide-specific genes can prove to be more rapid and cost-effective alternatives [26].

Since each surface polysaccharide structure is synthesized by a unique set of genes, it is often sufficient to identify just one or two polysaccharide processing genes and/or GTs to identify a serotype. Consequently, a molecular serotyping method based on the sequence diversities of genes in the OGCs may be better than the traditional serotyping system, although theoretically, both are dependent on the structural diversities of surface polysaccharide antigens to identify bacterial strains. As of now, several PCR-based molecular serotyping methods have been developed for specific genes to allow serotype identification in several bacteria such as *E*. *coli*, *Salmonella* spp., and *Shigella* spp. [27–29].

The Division of Enteric Pathogens (Central Public Health Laboratory, London) identifies 45 serogroups in *A*. *hydrophila* based on the diversity of this bacterium’s O antigens [30]. However, only four strains with confirmed serotypes have had the sequences and structures of their O antigens characterized (O11, O14, O18, and O34) [31–34].

In this study, the genomes of 14 *A*. *hydrophila* strains have been sequenced, and all putative OGCs have been identified and analyzed. Furthermore, we have identified 7 new gene cluster types from putative OGC sequences in 15 other *A*. *hydrophila* strains using genome sequences from the GenBank database. We have also developed and evaluated a Luminex bead-based suspension array that can rapidly detect *A*. *hydrophila* strains with high specificity and sensitivity. We believe that this array can be improved upon in the future through the addition of more serotype identifiers with newly designed primers and probes based on specific genes.

## Materials and methods

### Strains

The cultures for type strains of *A*. *hydrophila* were obtained from the Japan Collections of Microorganisms (JCM) and Division of Maricultural Organism Disease Control and Molecular Pathology of Yellow Sea Fisheries Research Institute (YSFRI). A list of all the 14 *A*. *hydrophila* strains used for whole genome sequencing in this study is provided in S1 Table.

### Genomic DNA extraction and sequencing

All *A*. *hydrophila* strains were maintained on tryptic soy broth (TSB) or tryptic soy agar (TSA) at 25 °C as previously described [35]. Genomic DNA was extracted using the Bacterial DNA Extraction Kit (CWBIO Co., Ltd, China) according to the manufacturer’s instructions. Whole genome sequencing of 14 *A*. *hydrophila* strains was performed with the Solexa paired-end sequencing technology (Illumina, Little Chesterford, Essex). The Solexa Genome Analyzer IIx (Illumina) was used to sequence each strain to obtain ~100-fold coverage. The Illumina reads were then assembled using the *de novo* assembly program Velvet (v2.2) to generate multi-contig draft genomes [36]. Gaps within the OGCs were filled using directed PCRs whose products were sequenced with BigDye terminator chemistry on ABI 3730 capillary sequencers.

BLAST and PSI-BLAST were used to search for and identify genes and proteins in the GenBank and Uniprot/SwissProt databases. TMHMM (v2.0) was used to identify potential transmembrane domains within protein sequences. MUSCLE (v3.8.31) was used for sequence alignment, and phyML v3.0 was used to construct maximum likelihood trees [37,38].

### Design methodology for probes and primers

Primer Premier (v5.0, Premier Biosoft International, Palo Alto, CA, USA) was used to design serotype-specific PCR primers based on DNA sequences of the processing genes *wzx*/*wzy* and *wzm*/*wzt* that were obtained in this study. The forward primer was biotinylated at the 5’-end to allow binding to the reporter dye streptavidin-R-phycoerythrin for detection on a Bio-Plex platform. The primers generated PCR fragments of 151–217 bp (S2 Table) that were then used to design serotype-specific probes based on the processing genes (S3 Table) using multiple-sequence alignments with MUSCLE (v3.8.31). The final probes were 18–25 bp in length, and synthesized with a 5’-end amino C-12 modification (AuGCT, China) and coupled to carboxylated beads (Bio-Rad Laboratories, Hercules, CA).

### Multiplex PCR amplification

A single multiplex PCR system was used to amplify the processing genes of 14 OGCs in a 50 μl reaction mixture consisting of 100 ng of genomic DNA, 1× Goldstar PCR buffer, 20 μM of each dNTP, 2.5 units of Goldstar DNA polymerase, 0.5 μM of each forward primer, and 2 μM of each reverse primer. The PCR reaction was carried out with an initial denaturation step at 94 °C for 5 min, followed by 34 cycles of the following conditions: 95 °C for 45 s (denaturation), 55 °C for 30 s (annealing), and 72 °C for 30 s (extension), and ended with a final extension step at 72 °C for 10 min. The PCR products were then directly used in hybridization reactions.

### Hybridization and Luminex analysis

The OGC probes were bound to different carboxylated beads as described previously [39]. Each working microsphere consisted of seven types of beads where each bead was coupled to a different OGC-specific probe. Hybridization was performed in a 50 μl mixture that included 17 μl of biotin-labeled PCR product and 33 μl of working microspheres; the mixture was subjected to 95 °C for 10 min for denaturation, and then incubated at 55 °C for 17 min in a thermal cycler. Of the three different temperatures (37 °C, 55 °C, and 60 °C) initially tested for probe hybridization, 55 °C was determined to be the optimal temperature for these assays according to the fluorescence signal intensity and stringency of hybridization obtained. The hybridization products formed were then transferred to a filter plate and washed three times with 1× Tetramethyl ammonium chloride (TMAC) buffer at 1,000 rpm for 1 min. For detection, 80 μl of streptavidin-R-phycoerythrin in 1× TMAC buffer was added to each well, followed by incubation at 53 °C for 20 min. Finally, the signals emitted by each set of beads were measured using a Bio-Plex 100 reader (BioRad) according to the manufacturer’s instructions. Data were analyzed using the Bio-Plex Manager 4.0, and all results are presented as median fluorescence intensity (MFI) values. The cut-off for a positive result was defined as a value that was three times greater than that of the mean MFI of the background.

In order to identify the 14 *A*. *hydrophila* strains used in this study, the suspension arrays were divided into 2 groups: group 1 (with *A*. *hydrophila* strains O7, O9, O10, O13, O16, O33, and O35), and group 2 (with *A*. *hydrophila* strains O19, O23, O24, O25, O29, O33, and O44). Array experiments were repeated three times for each group.

### Specificity and sensitivity detection of Luminex-based suspension array

To determine the sensitivity of the suspension array, serial ten-fold gradient dilution of the genomic DNA of O7 and O23 comprising 0.1 ng/μL, 1.0 ng/μL, 10 ng/μL to 100 ng/μL were prepared, and 1μL of each dilution was used as the template for multiplex PCR amplification and hybridization of the suspension array for the sensitivity test.

### Identification of OGCs from genome sequences

Previously published genome and OGC sequences of 15 *A*. *hydrophila* strains other than those used for whole genome sequencing in this study were downloaded from GenBank (S4 Table). We then used BLASTP to extract putative OGC sequences from whole genome sequences. A database containing the newly sequenced *A*. *hydrophila* OGC sequences and the previously published OGC sequences was set up, and analyzed. Gene clusters sharing high-level identity (>97%) and possessing the same gene organization were classified as belonging to the same serotype.

## Results

### Sequence analysis of putative O antigen gene clusters of *A*. *hydrophila*

**Fig 1:**
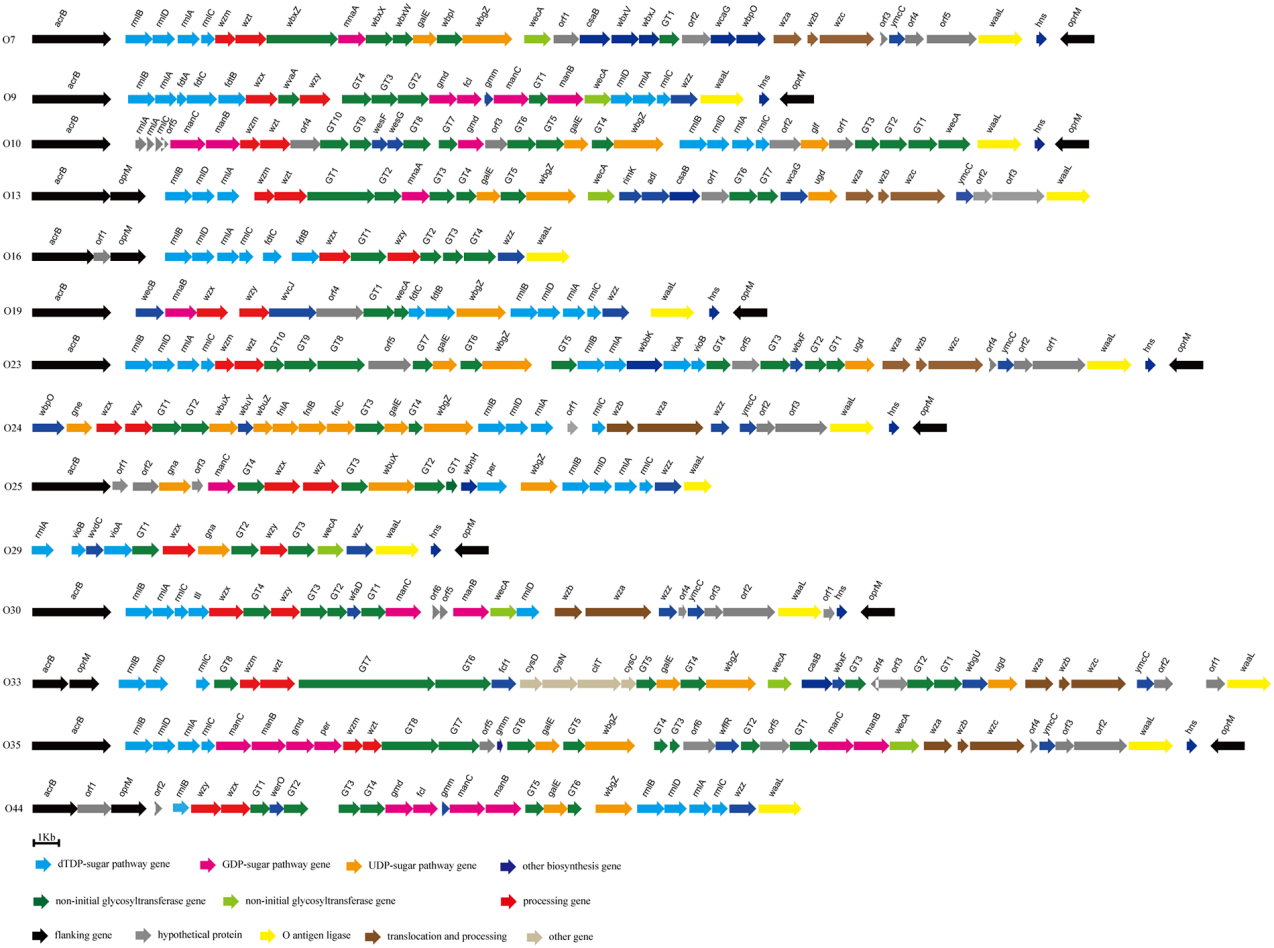
O antigen gene clusters from the 14 *A*. *hydrophila* serotypes sequenced in this study. The sequences of the 14 *A*. *hydrophila* O antigen gene clusters have been deposited in the GenBank database with accession numbers MH449673 to MH449686.

In this study, the genomes of 14 *A*. *hydrophila* strains that were sequenced included those of 12 strains (O7, O9, O10, O13, O19, O24, O25, O29, O30, O33, O35, and O44) from JCM, and two strains (O16 and O23) from YSFRI (S1 Table). Putative OGC regions were located downstream of the housekeeping genes *acrB* and/or *oprM* (Fig 1), which are conserved and encode for a multidrug efflux pump subunit and outer membrane protein, respectively. The newly sequenced *A*. *hydrophila* OGCs ranged in size from 15,777–43,887 bp, and encoded 15–38 ORFs. The average GC content of all these OGCs is ~45%, which is significantly lower than the average GC content of the *A*. *hydrophila* genome as a whole (61%). The allocation and accession numbers for the OGCs identified in this study are summarized in S1 Table. We have also observed and documented some unusual features of the *A*. *hydrophila* OGCs identified in this study, such as, the occurrence of the initial glycosyltransferase gene *wecA* within the OGCs; in most *Enterobacteriaceae*, *wecA* is not present within the OGC. Since variations such as these often indicate the occurrence of recent genetic changes, it is likely that the O antigen forms of *A*. *hydrophila* documented in this study may have evolved recently.

### Nucleotide sugar biosynthesis genes

**Fig 2:**
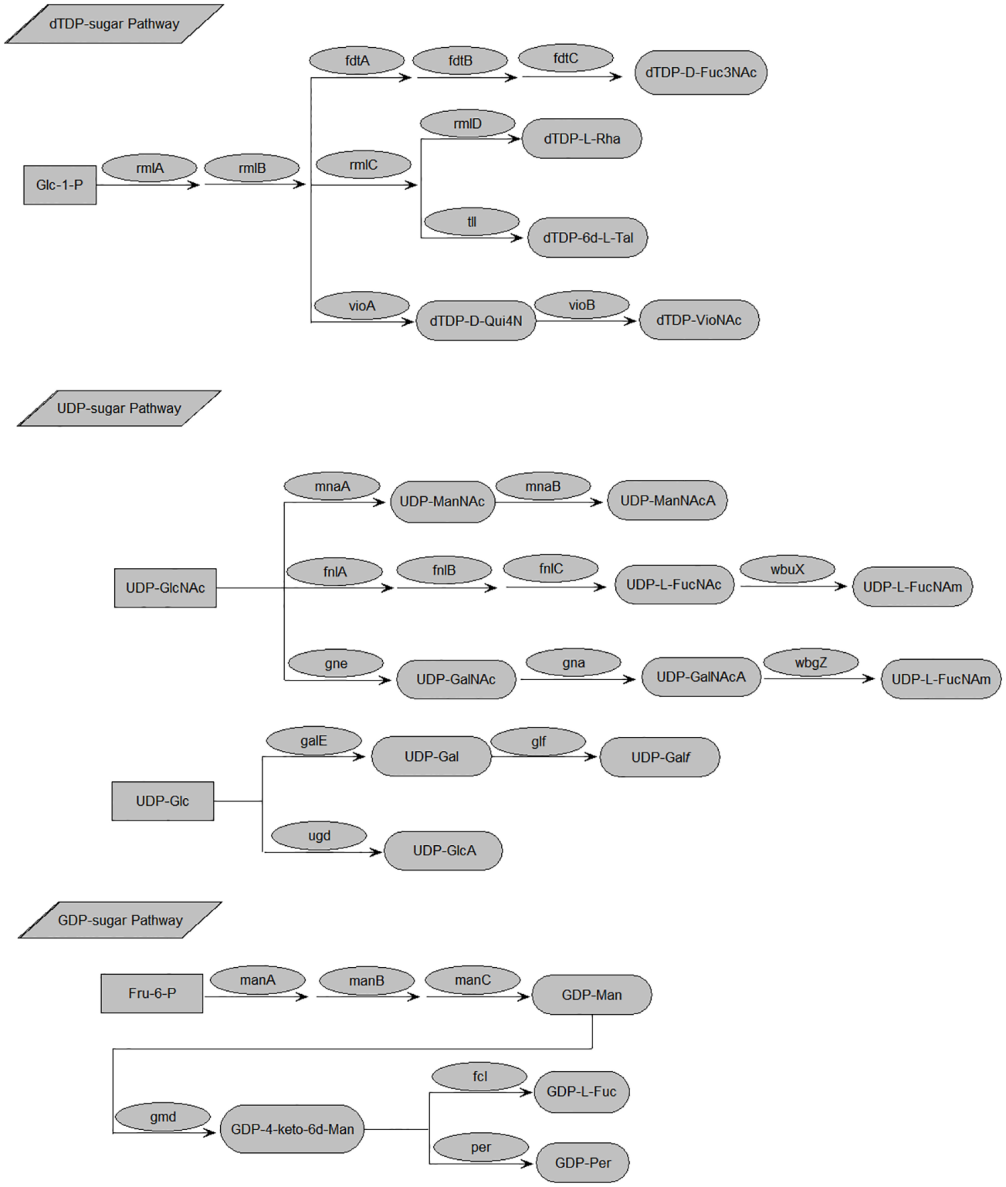
Biosynthesis pathways for putative rare sugars in *A*.*hydrophila* O anigens. ugd, UDP-glucose 6 dehydrogenase[41]; glf, UDP-galctopyranose mutase[42]; galE, UDP-glucose-4-epimerase[43]; gne, UDP-N-acetylglucosamine-4-epimerase[44]; gna, UDP-GalNAcA synthetase [45]; wbgZ, C-5 epimerase[46]; fnlA, 4,6-dehydratase, 3- and 5-epimerase; fnlB, reductase; fnlC, C-2 epimerase [47]; wbgX, UDP-D-FucNAc4N synthetase[48]; mnaA, UDP-N-acetylglucosamine-2-epimerase[49]; mnaB, UDP-ManNAc dehydrogenase[50]; rmlA, glucose-1-phosphate thymidylyltransferase[51]; rmlB, dTDP-D-glucose 4,6-dehydratase[52]; rmlC, dTDP-4-keto-6-deoxy-Dglucose 3,5-epimerase[53]; rmlD, dTDP-6-deoxy-L-mannose-dehydrogenase [54]; manA, phosphomannose isomerase; manB, phosphomannomutase; manC, mannose-1-phosphate guanylyltransferase[43]; gmd, GDP-mannose-4,6-dehydratase [55]; fcl, GDP-L-fucose synthetase[56]; vioA, aminotransferase[57]; vioB, dTDP-4-amino-4,6-dideoxy-D-glucose acyltransferase[58]; fdtA, dTDP-6-deoxy-hex-4-ulose isomerase; fdtB, dTDP-6-deoxy-D-xylo-hex-3-ulose aminase; fdtC, dTDP-D-Fuc3N acetylase[59]; per, GDP-4-keto-6-deoxy- d-mannose-3-dehydratase[60]; tll, dTDP-6-deoxy-L-lyxo-4-hexulose reductases[61].

Genes involved in synthesizing nucleotide precursors of common sugars (UDP-D-Gal, UDP-D-Glc, and UDP-D-GlcNAc) are usually not located within OGCs. However, one gene which was highly homologous to *galE*, (an enzyme that converts UDP-D-Glc to UDP-D-Gal) has been found in several *A*. *hydrophila* OGCs (O7, O23, O24, O33, O35, and O44). In most of the *A*. *hydrophila* OGCs identified in this study, *rmlABCD* (which is responsible for dTDP-L-Rha synthesis) [40], *rmlABC/tll* (for dTDP-6d-L—Tal synthesis), *rmlAB/fdtABC* (for dTDP-D-Fuc4NAc synthesis), *manABC* (for GDP-D-Man synthesis), *fnlABC/wbuX* (for UDP-L-FucNAc and UDP-L-FucNAm synthesis), *manABC/gmd/per* (for GDP-Per synthesis), and *rmlAB/vioAB* (for dTDP-VioNAc synthesis), were also found to be present. Biosynthetic pathways for all putative rare sugars present in *A*. *hydrophila* O antigens are shown in Fig 2.

### Glycosyltransferase genes

The gene *wecA*, which is responsible for initiating O antigen synthesis, and transfers the first sugar residue (GalNAc or GlcNAc) during the process [62], is usually located outside the OGC in *E*. *coli*, *Salmonella* spp., and *Shigella* spp. [63]. However, in most *A*. *hydrophila* OGCs, a homologue of *wecA* was found inside the gene cluster. Since published structures of O antigens from *A*. *hydrophila* contain GalNAc or GlcNAc [31,64], it is likely that a *wecA* homologue initiates the synthesis of O antigens in *A*. *hydrophila* strains, in a process similar to what occurs in *E*. *coli*, *Salmonella* spp., and *Shigella* spp. Each OGC identified in this study has also been observed to possess non-initial glycosyltransferase genes and oligosaccharide unit processing genes. The characteristics of all open reading frames (ORFs) detected in every putative OGCs identified in this study are summarized in S5 Table.

Since the GTs responsible for linkages between sugars in the repeat units of polysaccharides are highly diverse in terms of sequence, and combinations of different donor sugars, acceptor sugars, and linkage types can vary widely, the specificity of a GT for particular combinations are rarely confirmed experimentally. Despite this drawback, closely related GTs can be associated with specific linkage classes, and realistic predictions can be made for the functions of all or most GTs in a gene cluster. In this study, each *A*. *hydrophila* OGC contained 2–11 putative GT genes. In all, our study has identified a total of 91 GT genes from 15 OGCs. Of these, 39 GTs have been classified into 15 homology groups using the software OrthoMCL (v2.0); each homology group contains at least 2 GTs (S6 Table). As the grouping is based on similarities in protein sequences, all GTs in the same homology group are considered to have similar functions.

### O antigen processing genes

**Fig 3:**
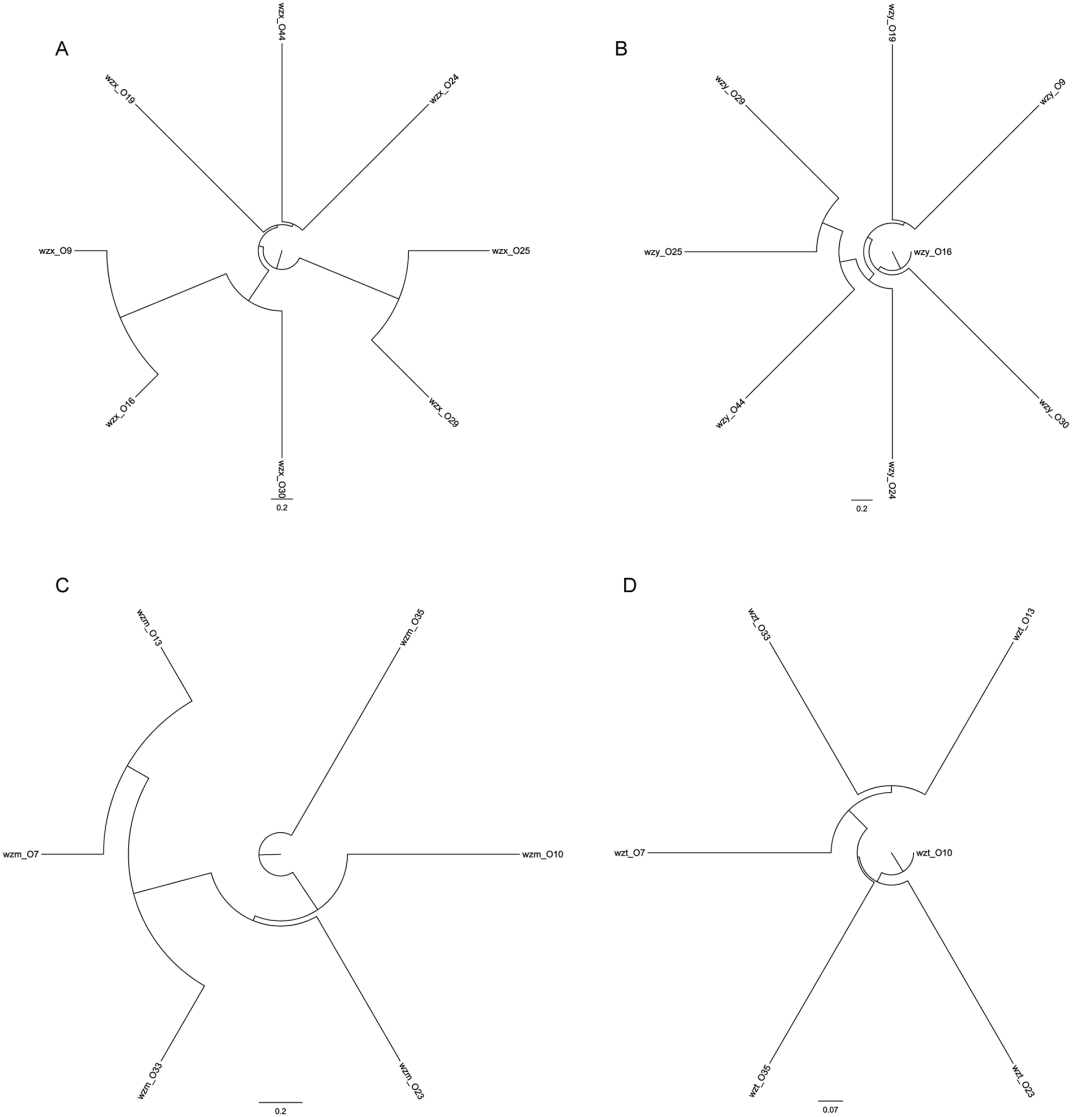
Phylogenetic trees constructed from the sequences of processing genes from the 14 *A*. *hydrophila* serotypes used in this study. The *wzx* (A), *wzy* (B), *wzm* (C), and *wzt* (D) trees were constructed using *wzx*, *wzy*, *wzm*, and *wzt* gene sequences. The sequences were aligned using MUSCLE (v3.8), and the trees were constructed using phyML (v3.0).

The OGCs in *A*. *hydrophila* are known to contain both synthesis and translocation pathways, namely, the Wzx/Wzy pathway and the ATP-binding cassette (ABC) transporter pathway (*wzm/wzt* genes), respectively. Amongst the 14 newly sequenced OGCs in this study, 8 (O9, O16, O19, O24, O25, O29, O30, and O44) contained *wzx*/*wzy* genes, while the other 6 (O7, O10, O13, O23, O33, and O35) contained *wzm*/*wzt* genes. We identified distinctive forms of *wzx*/*wzy* and *wzm*/*wzt* genes using unique serial numbers that were based on homology groups constructed using OrthoMCL (v.2.0). Based on the sequence alignments for each gene, the most homologous pair of *wzx* was 58.10%, and the values of *wzy*, *wzm* and *wzt* were 69.30%, 61.70% and 56.60% respectively. The diversity of processing genes provided us with the opportunity to apply molecular techniques to identify and classify different serotypes with the aim of developing a process that can be used to diagnose *A*. *hydrophila* infections. As expected, *wzx* genes were found to encode proteins with 10–12 transmembrane segments (TMS), *wzy* genes encoded proteins with 9–12 TMS, and the *wzm* and *wzt* genes encoded for proteins with at least 5 TMS. Four phylogenetic trees were constructed using the homology groups created with the sequences of these processing genes (Fig 3). We utilized the high diversity exhibited by these processing genes to develop a molecular tool to identify and classify different *A*. *hydrophila* serotypes.

### Additional genes identified

A putative O antigen ligase, *waaL*, was found in all *A*. *hydrophila* OGCs identified in this study; the *waaL* gene is known to be responsible for encoding a protein that mediates the ligation of pre-assembled O antigens to the lipid A-core moieties, and plays an important role in the biosynthesis of integral LPS [65].

Besides the *waaL* gene, several *A*. *hydrophila* OGCs (O7, O9, O10, O19, O23, O24, O29, O30 and O35) contained a gene that was highly homologous (identity ~100%) to the histone-like nucleoid structuring protein (*hns*) in *Aeromonas veronii* B565, and were found to be located close to the 3’-end of the housekeeping gene *oprM*.

Another common feature of the putative OGCs identified in this study on *A*. *hydrophila*, is the presence of a conserved block of homologues of the *wza*, *wzb*, and *wzc* genes, which have been reported to occur in the K antigen synthesis clusters of *E*.*coli* group 1 bacteria [66]. While the *wzb* gene is essential for the maintenance of both polymerization and capsular polysaccharide (CPS) export, *wzc* encodes an integral inner-membrane tyrosine autokinase, and *wza* codes for an outer-membrane protein that forms a complex with the protein encoded by *wzc*. In contrast to the O antigen processing system, CPS export is coupled to Wzx/Wzy-dependent polymerization, and mutations in the *wza* or *wzc* genes can result in similar acapsular phenotypes.

We have also identified a *wzz* gene homologue, which in 8 *A*. *hydrophila* OGCs (O9, O16, O19, O24, O25, O29, O30 and O44), is responsible for determining the O antigen chain length. Furthermore, we have identified a homologue of the *ymcC* gene in several OGCs (O7, O13, O23, O24, O30, O33 and O35)that encodes for a protein involved in extracellular polysaccharide production [67].

The O33 gene cluster was separated into two sections by four genes involved in the biosynthesis of sulfates (*cysC*, *citT*, *cysN*, and *cysD*)[68].

In all, our results and data indicate that OGCs in *A*. *hydrophila* are located in the genetic region downstream of *acrB* and/or *oprM*, and that the genetic diversity of this region could be used to develop a molecular serotyping system.

### PCR-based suspension arrays for molecular detection of O serotypes of 14 different *A*. *hydrophila*

**Fig 4:**
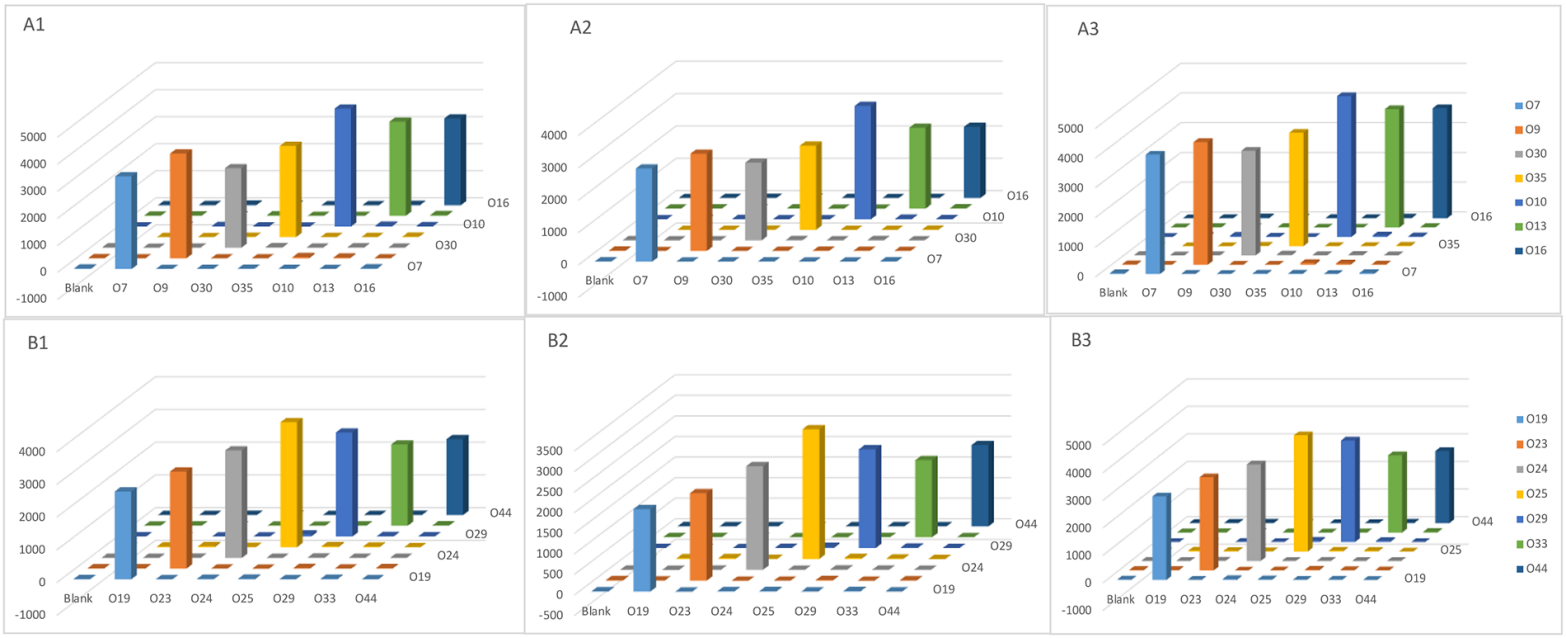
The hybridization results of the 14 *A*. *hydrophila* strains. The suspension arrays were divided into 2 groups (A) O7, O9, O30, O35, O10, O13, and O16; (B) O19, O23, O24, O25, O29, O33, and O44. No cross reactions were observed for any probe tested in this study, and the ‘Blank’ indicates a negative control. The x-axis represents the PCR products of different serotypes, the y-axis represents the MFI values, and the z-axis represents the specific probes used for detection.

Amongst the three gene classes in the OGCs of *A*. *hydrophila*, the processing genes and GTs exhibit higher molecular diversity than the nucleotide sugar precursor synthesis genes, and therefore, these genes have greater potential as genetic targets in developing a molecular identification system for *A*. *hydrophila* serotypes [40]. In this study, the processing genes showed low sequence identities ranged from 22.30% to 58.10%, indicating that these genes could be specific for different serotypes, and so can be highly useful in molecular serotyping. We have therefore developed a PCR-based suspension array using the processing genes of the OGCs from *A*. *hydrophila* for molecular serotyping of these bacteria (Fig 4).

A total of 30 *A*. *hydrophila* strains known to carry the 14 newly sequenced OGCs along with other pathogenic bacteria, including *Salmonella* spp. (n = 1), *Shigella* spp. (n = 1), *E*. *coli* (n = 1), *Klebsiella pneumonia* (n = 2), and *Vibrio cholera* (n = 2), were used to test the specificity of the multiplex Luminex-based array (S1 Table). No non-specific amplicons in the multiplex PCR products were observed. Each serotype-specific probe detected homologous strains correctly in a series of Luminex-based array analyses. Heterologous signals corresponding to other pathogenic bacteria were also not observed. The S/B ratios of each probe that tested against its homologous DNA were significantly higher than for those against non-homologous DNA. The S/B ratios of the positive samples ranged from 2.0–5.0, and no cross reactions were observed for any of the probes tested.

To determine the sensitivity of the suspension array, a 10-fold serial dilution experiment was conducted (100.0 ng to 0.1 ng of genomic DNA) using the O7 and O23 strains. Based on the positive signals generated, the sensitivity of the assay using genomic DNA was 0.1ng. The remaining 12 serogroups strains were identified using 0.1ng genomic DNA, suggesting that this was the minimal dose needed for detection.

### Molecular serotyping of *A*. *hydrophila* strains based on genome sequences

**Fig 5:**
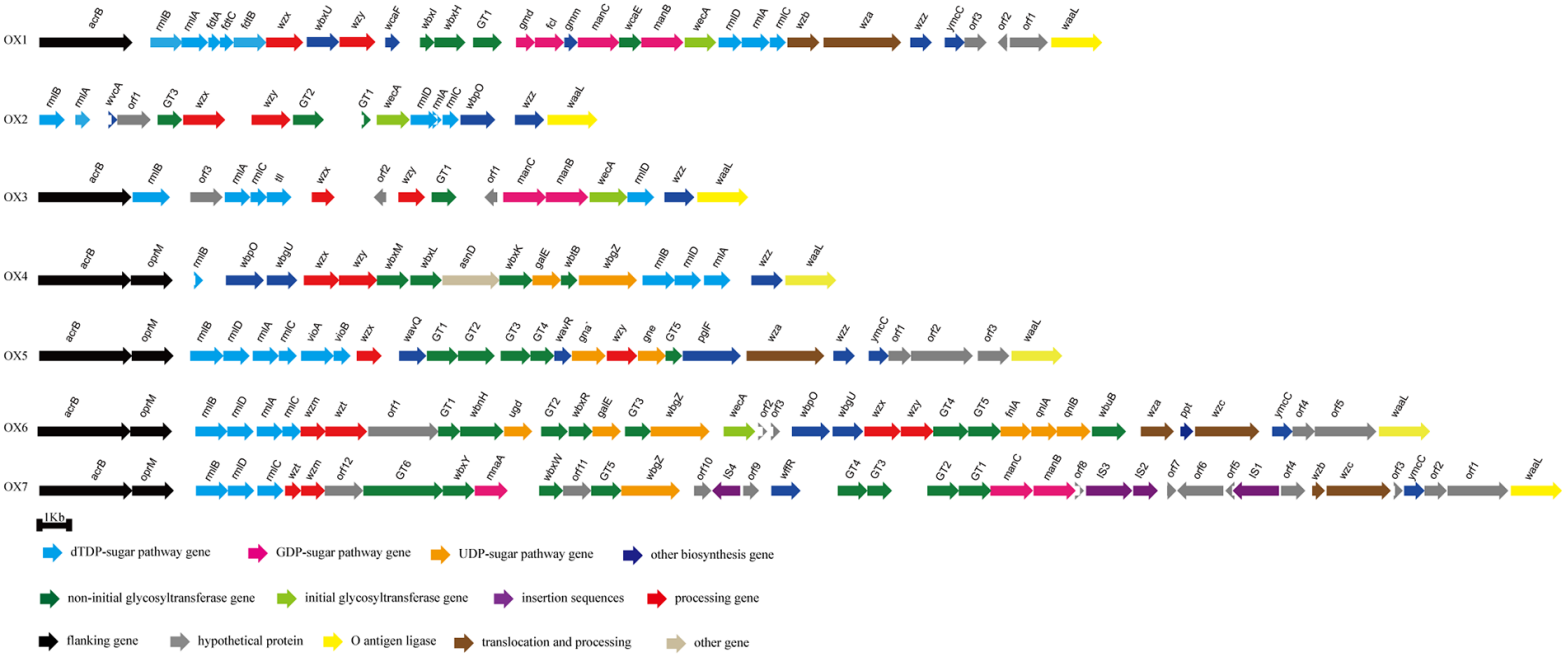
Seven novel putative OGSs that were identified in 15 strains whose genomes were publicly available. Genes are represented by arrows and colored according to the gene key at the bottom with gene names indicated above each arrow.

Details of the 15 *A*. *hydrophila* genomes downloaded from the GenBank database used to extract putative OGCs are provided in S4 Table. The 14 OGCs newly sequenced in this study along with 6 more previously published sequences were used to set up a special *A*. *hydrophila* OGC database for identifying putative OGCs from the downloaded genomic data. We initially identified 13 putative OGC sequences using the database, but had to discard 6 of these sequences due to duplications; we were finally able to identify 7 unique OGCs through this analysis (Fig 5). In all, our combined analysis has yielded a total of 25 unique OGCs that can be used to identify *A*. *hydrophila* serotypes via molecular serotyping (S1 Fig).

Our analysis has also brought to light several variations in the 25 OGCs that we have analyzed. Interestingly, the *wzm*/*wzt* and *wzx*/*wzy* genes were found to be located within the same gene cluster in OX6. To our knowledge, this is the first instance where the genes for these two distinct pathways have been found to occur in one cluster. We suppose that the O and K antigens cluster are both located in this region.

We have also discovered the existence of 4 insertion sequences (IS) in the OGC of OX7. Several IS elements are known to play important roles in the evolution of bacterial genomes as they can activate, inactivate, and translocate genetic sequences. We believe that the 4 IS elements in OX7 may be involved in the formation of new O antigen forms.

## Discussion

Isolates of *A*. *hydrophila* from clinical and environmental samples are known to cause various conditions such as gastroenteritis, diarrhea, septicemia, and urinary tract infections[4,5]. Since serotypes of virulent strains of *A*. *hydrophila* often express O antigens on their surfaces, the OGC is thought to be an important virulence factor that contributes to the pathogenicity of this organism [3]. As of now, 45 serotypes of *A*. *hydrophila* have been identified [30], although molecular serotype data for this species is still lacking. Furthermore only 4 verified O antigen types from *A*. *hydrophila* have been sequenced and/or structurally characterized (O11, O14, O18, and O34) as most of the studies on this organism have focused mainly on strain type, and not serotype [69]. In this study, we have identified 14 new OGCs in *A*. *hydrophila*, most of which are located downstream of *acrB* and/or *oprM*. By including data from previously published *A*. *hydrophila* genomes, we have identified a total of 25 distinct O serotypes for this organism. This, we believe provides a good base for establishing an assay for the molecular serotyping of *A*. *hydrophila*.

Serotyping, or serology, is a subtyping test that is based on detecting differences in bacterial surfaces. The gold standard in serotyping uses O antigen-specific antisera for the identification of different pathogens or strains of pathogens. Most strains, especially pathogenic ones, are often referred to by their serotypes—an example being *A*. *hydrophila* O34, which is the single most common *Aeromonas* serotype that causes several types of infections in humans [70]. Since many O-antigen based bacterial serotypes are associated with specific disease conditions such as meningitis, systematic infection, diarrhea, etc., serotyping is an invaluable tool for epidemiological investigations. However, there are many problems associated with traditional serotyping. Apart from being labor-intensive and time consuming, cross reactivity, and unavailability of standard antisera can cause problems in serotype identification. In addition, this method cannot identify ‘rough strains’ which are isolates that lack surface antigens.

Faster, and more cost-effective alternatives to conventional serotyping can be devised using DNA-based typing methods based on polysaccharide-specific genes. Molecular serotyping methods, such as pulsed-field gel electrophoresis (PFGE), multilocus sequence typing (MLST), multiplex PCR, etc., can be used to simultaneously detect several specific genes responsible for the synthesis of O or K antigens. Since the rapid development of next-generation sequencing technologies make it possible to perform routine whole genome sequencing of pathogens at relatively rapid rates and affordable costs, several tools based on whole genome sequencing and *in silico* serotyping have also been developed. Of these, the Luminex-based array system is a multiplex microsphere-based suspension system that offers a promising molecular diagnostic platform for the development of a high-throughput system to simultaneously detect hundreds of targets in protein and nucleic acid studies.

Using whole genome sequencing and *in silico* analyses, we have identified a total of 25 putative OGCs from *A*. *hydrophila*, which we have used to develop a molecular serotyping tool for this organism. However, the detection range of our assay system needs to be extended with more isolates in the future, as we were only able to test the system on the 14 OGC forms that were available to us. And also, the *hns* gene is thought to play an important role in DNA condensation and may be a key regulator of gene expression in response to environmental changes [71]. In *Vibrio cholera*, the *hns* gene is a repressor of exopolysaccharide biosynthesis genes and biofilm formation [72]. The *hns* gene homologs identified here may play the same role in *A*. *hydrophila*, and require further investigation in the future. Furthermore, a more detailed study of each O antigen and its polysaccharide structure is necessary for a better understanding of the genetics and evolution of the O antigens of *A*. *hydrophila*.

## Supporting information

S1 Table The *Aeromonas hydrophila* strains and other bacterial strains used in this study. JCM- Japan Collection of Microorganisms, Japan; YSFRI -Yellow Sea Fisheries Research Institute, Chinese Academy of Fishery Sciences, China; ATCC: American type culture collection; CDC: Centers for Disease Control, China; CMCC: National Center for Medical Culture Collections, China.
(DOC)

S2 Table The primers used in this study. (DOCX)

S3 Table The probes used in this study. (DOC)

S4 Table The genomes and OGCs download from GenBank. (DOC)

S5 Table Characteristics of the ORFs in all putative OGCs. (DOC)

S6 Table The homologous groups of glycosyltransferases in *Aeromonas hydrophila* OGC. (DOC)

S1 Fig Phylogenetic trees constructed from the sequences of processing genes from the 25 *A*. *hydrophila* serotypes used in this study. The *wzx* (A), *wzy* (B), *wzm* (C), and *wzt* (D) trees were constructed using *wzx*, *wzy*, *wzm*, and *wzt* gene sequences. The sequences were aligned using MUSCLE (v3.8), and the trees were constructed using phyML (v3.0).
(TIF)
