# Supplementary material for: Identifying genetic diversity of O antigens in Aeromonas hydrophila for molecular serotype detection
Source: PLoS One. 2018 Sep 5;13(9):e0203445. doi: 10.1371/journal.pone.0203445 (PMC6124807; doi:10.1371/journal.pone.0203445)
Supplement: S1 Table — JCM- Japan Collection of Microorganisms, Japan; YSFRI -Yellow Sea Fisheries Research Institute, Chinese Academy of Fishery Sciences, China; ATCC: American type culture collection; CDC: Centers for Disease Control, China; CMCC: National Center for Medical Culture Collections, China. (DOC) [file pone.0203445.s001.doc]

**S1 Table. The *Aeromonas hydrophila* strains and other bacterial strains used in this study**

| Serotype | Strain | Species | Source | Accession Number |
| --- | --- | --- | --- | --- |
| O7 | G5369 | *A. hydrophila* | JCM^1^ | MH449673 |
| O9 | G5371 | *A. hydrophila* | JCM | MH449674 |
| O10 | G5372 | *A. hydrophila* | JCM | MH449675 |
| O13 | G5374 | *A. hydrophila* | JCM | MH449676 |
| O16 | G5181 | *A. hydrophila* | YSFRI^2^ | MH449677 |
| O19 | G5376 | *A. hydrophila* | JCM | MH449678 |
| O23 | G5183 | *A. hydrophila* | YSFRI | MH449679 |
| O24 | G5380 | *A. hydrophila* | JCM | MH449680 |
| O25 | G5381 | *A. hydrophila* | JCM | MH449681 |
| O29 | G5385 | *A. hydrophila* | JCM | MH449682 |
| O30 | G5386 | *A. hydrophila* | JCM | MH449683 |
| O33 | G5388 | *A. hydrophila* | JCM | MH449684 |
| O35 | G5400 | *A. hydrophila* | JCM | MH449685 |
| O44 | G5391 | *A. hydrophila* | JCM | MH449686 |
| Other strains used for Luminex-based array analyses | | | | |
| O2 | G5365 | *A. hydrophila* | JCM |  |
| O4 | G5366 | *A. hydrophila* | JCM |  |
| O5 | G5367 | *A. hydrophila* | JCM |  |
| O8 | G5370 | *A. hydrophila* | JCM |  |
| O20 | G5377 | *A. hydrophila* | JCM |  |
| O21 | G5378 | *A. hydrophila* | JCM |  |
| O26 | G5382 | *A. hydrophila* | JCM |  |
| O27 | G5383 | *A. hydrophila* | JCM |  |
| O32 | G5387 | *A. hydrophila* | JCM |  |
| 2a | G2555 | *Shigella flexneri*2a | ATCC^3^ |  |
| O157 | G2735 | *E.coli* O157:H7 | CDC^4^ |  |
| 9 | G5339 | *Salmonella typhi*9 | CDC |  |
|  | G1743 | *Klebsiella pneumoniae* | CMCC^5^ |  |
|  | G1744 | *Klebsiella pneumoniae* | CMCC |  |
|  | G2709 | *Vibrio cholerae* | CMCC |  |
|  | G2710 | *Vibrio cholerae* | CMCC |  |

^1^JCM: Japan Collection of Microorganisms

^2^YSFRI: Yellow Sea Fisheries Research Institute, Chinese Academy of Fishery Sciences(CAFS)

^3^ATCC: American type culture collection

^4^CDC: Centers for Disease Control, China

^5^CMCC：National Center for Medical Culture Collections, China
