# Supplementary material for: Identifying genetic diversity of O antigens in Aeromonas hydrophila for molecular serotype detection
Source: PLoS One. 2018 Sep 5;13(9):e0203445. doi: 10.1371/journal.pone.0203445 (PMC6124807; doi:10.1371/journal.pone.0203445)
Supplement: S2 Table — (DOCX) [file pone.0203445.s002.docx]

**S2 Table. The primers used in this study**

| **Serotype** | **Target gene** | **Lab primer No.** | **F/R^1^** | **Primers（5'-3')** | **Product Length（bp）** |
| --- | --- | --- | --- | --- | --- |
| O7 | *wzm* | wl69741 | F | GCTTATGTTTCTCTCTCCACTA | 151 |
|  | *wzm* | wl69742 | R | TTCCCCACATGCCCCACTCT | 151 |
| O9 | *wzx* | wl69745 | F | TTGGCGTGAAGATGTCCGAG | 159 |
|  | *wzx* | wl69746 | R | TGCAAGCGTCACCGGCCAAA | 159 |
| O10 | *wzm* | wl69751 | F | ATTTTCCCGGTTTACCCTATTT | 203 |
|  | *wzm* | wl69752 | R | ATGGCTTAATCGCCGCGGG | 203 |
| O13 | *wzm* | wl69753 | F | TGTTAAAAACTGGAATCTTATT | 165 |
|  | *wzm* | wl69754 | R | ACCTCCAGACCATCTAGCTT | 165 |
| O16 | *wzx* | wl69757 | F | TTACATGGAGCGTTAATAGCTG | 171 |
|  | *wzx* | wl69758 | R | GAGCAGAAGTCACTGCCATCAG | 171 |
| O19 | *wzx* | wl69759 | F | TGCGGTTCTAAGTGCGGCGA | 152 |
|  | *wzx* | wl69760 | R | TTCTTGACAGGAGGGTGAAAGA | 152 |
| O23 | *wzm* | wl69761 | F | TCTTATATAAAAGATCTAGGCA | 157 |
|  | *wzm* | wl69762 | R | ATTTTACGGGCCCCTGAAATAA | 157 |
| O24 | *wzy* | wl69819 | F | CCCTGAATTAGGGAAGATTCGT | 194 |
|  | *wzy* | wl69820 | R | CGTACCAGCACAATCGCAAC | 194 |
| O25 | *wzy* | wl69821 | F | ATGCCTACACTGAGGCTCGG | 180 |
|  | *wzy* | wl69822 | R | CCCTTGACCCAACCGGCCTT | 180 |
| O29 | *wzx* | wl69825 | F | CTTGGAATACTCGTAGTGAATG | 165 |
|  | *wzx* | wl69826 | R | CTATAACTACTCCATCGACTTA | 165 |
| O30 | *wzx* | wl69747 | F | GTTGGGATTGGGCAGAATTTCC | 217 |
|  | *wzx* | wl69748 | R | AGCTTAGTACCCGCTTGAATTG | 217 |
| O33 | *wzm* | wl69827 | F | ATTTAGCGAAGTGTTCAAGGCT | 198 |
|  | *wzm* | wl69828 | R | AAGATTGACAAAAGGGAGTATT | 198 |
| O35 | *wzt* | wl69749 | F | ACATCAAGCTTAAATTTGATGT | 189 |
|  | *wzt* | wl69750 | R | AAAAAAGCAAAAATACGATCGG | 189 |
| O44 | *wzx* | wl69831 | F | CAGAAGCGGAAGTTCAGTTGTT | 151 |
|  | *wzx* | wl69832 | R | ACCGAATGGTAGCGGGAACC | 151 |

^1^Forward or Reverse direction
