# Supplementary material for: Identifying genetic diversity of O antigens in Aeromonas hydrophila for molecular serotype detection
Source: PLoS One. 2018 Sep 5;13(9):e0203445. doi: 10.1371/journal.pone.0203445 (PMC6124807; doi:10.1371/journal.pone.0203445)
Supplement: S3 Table — (DOC) [file pone.0203445.s003.doc]

**S3 Table. The probes used in this study**

| **Serotype** | **Target gene** | **Probe No.** | **Primers（5'-3')** |
| --- | --- | --- | --- |
| O7 | *wzm* | OA4969 | NH_2_C_12_CATACGCAATAGAGACGGCTAGAGG |
| O9 | *wzm* | OA4971 | NH_2_C_12_TGCGTGTAGGTGGCATCAGCAATCG |
| O10 | *wzm* | OA4974 | NH_2_C_12_GCTTGGGGATGACACTCGGAGTACT |
| O13 | *wzm* | OA4975 | NH_2_C_12_AAGGTTCCTTGATCGGAGTCCTGTG |
| O16 | *wzx* | OA4977 | NH_2_C_12_ATACACCGTAAGGCAATAGGTGGCT |
| O19 | *wzx* | OA4978 | NH_2_C_12_CAAAGACGTGGTAGGGACGATATTA |
| O23 | *wzm* | OA4979 | NH_2_C_12_AGGTTAGCCCCATCTTCGAAAGTGT |
| O24 | *wzy* | OA4980 | NH_2_C_12_ATGATCGACTCACCGGAAGAACCGT |
| O25 | *wzy* | OA4981 | NH_2_C_12_CGATCACCCCCACAACGAGTTCCTC |
| O29 | *wzx* | OA4983 | NH_2_C_12_GAATGACCTCACTGGATAGAATCCT |
| O30 | *wzx* | OA4972 | NH_2_C_12_AGTGGTACCGTAATAACTGCTAATG |
| O33 | *wzm* | OA4984 | NH_2_C_12_TGCGCCCGGATTGATACTAGGAAAT |
| O35 | *wzy* | OA4973 | NH_2_C_12_TTAGGTCAATACATGACAGCGCATG |
| O44 | *wzx* | OA4986 | NH_2_C_12_TTGCTTCACTGATGCGACTTGTTTC |
