# Supplementary material for: Identifying genetic diversity of O antigens in Aeromonas hydrophila for molecular serotype detection
Source: PLoS One. 2018 Sep 5;13(9):e0203445. doi: 10.1371/journal.pone.0203445 (PMC6124807; doi:10.1371/journal.pone.0203445)
Supplement: S4 Table — (DOC) [file pone.0203445.s004.doc]

**S4 Table. The genomes and OGCs download from GenBank**

| **Species name** | **Strains** | **Accession Number** | **Putative O serotype** | **Position** | **Processing genes** | **Identity** |
| --- | --- | --- | --- | --- | --- | --- |
| **Genomes** | | | |  |  |  |
| *A. hydrophila* | ML09-119 | CP005966.1 | OX1 | 3497604.. 3533648 | wzx/wzy |  |
| *A. hydrophila* | 4AK4 | CP006579.1 | OX2 | 2913725.. 2932650 | wzx/wzy |  |
| *A. hydrophila* | YL17 | CP007518.2 | OX3 | 4319024.. 4343094 | wzx/wzy |  |
| *A. hydrophila* | AL06-06 | CP010947.1 | OX4 | 3314895.. 3341961 | wzx/wzy |  |
| *A. hydrophila* | AH10 | CP011100.1 | OX5 | 1564769.. 1599467 | wzx/wzy |  |
| *A. hydrophila* | GYK1 | CP016392.1 | OX6 | 1512634.. 1559863 | wzm/wzt+wzx/y |  |
| *A. hydrophila* | WCHAH045096 | CP028568.1 | OX7 | 3418778.. 3470421 | wzm/wzt |  |
| *A. hydrophila* | AL09-71 | CP007566.1 | OX1 | 3497129.. 3533173 | wzx/wzy | 100% |
| *A. hydrophila* | pc104A | CP007576.1 | OX1 | 3497101.. 3533145 | wzx/wzy | 100% |
| *A. hydrophila* | J-1 | CP006883.1 | O33 | 1484847.. 1532077 | wzm/wzt | 98% |
| *A. hydrophila* | ATCC 7966 | CP000462.1 | OX6 | 3225401.. 3273316 | wzm/wzt+wzx/y | 98% |
| *A. hydrophila* | NJ-35 | CP006870.1 | OX6 | 1483842.. 1531075 | wzm/wzt+wzx/y | 99% |
| *A. hydrophila* | JBN2301 | CP013178.1 | OX1 | 1558017.. 1594061 | wzx/wzy | 99% |
| *A. hydrophila* | D4 | CP013965.1 | OX1 | 1556909.. 1592953 | wzx/wzy | 99% |
| *A. hydrophila* | AHNIH1 | CP016380.1 | O25 | 1633914.. 1662840 | wzx/wzy | 98% |
| **Published OGCs** | | | |  |  |  |
| *A. hydrophila* | PPD134/91 | AF148126.2 | O18 |  | wzx/wzy |  |
| *A. hydrophila* | ZC1 | KF647894.1 | OX1 |  | wzx/wzy | 99% |
| *A. hydrophila* | S04-690 | KF647893.1 | OX1 |  | wzx/wzy | 100% |
| *A. hydrophila* | AL06-06 | KC999971.1 | OX4 |  | wzx/wzy | 99% |
| *A. hydrophila* | PB10-118 | KC999977.1 | OX1 |  | wzx/wzy | 100% |
| *A. hydrophila* | AL10-121 | KC999976.1 | OX1 |  | wzx/wzy | 100% |
| *A. hydrophila* | ML09-122 | KC999975.1 | OX1 |  | wzx/wzy | 100% |
| *A. hydrophila* | ML09-121 | KC999974.1 | OX1 |  | wzx/wzy | 100% |
| *A. hydrophila* | ML09-119 | KC999973.1 | OX1 |  | wzx/wzy | 100% |
| *A. hydrophila* | AL09-79 | KC999972.1 | OX1 |  | wzx/wzy | 100% |
| *A. hydrophila* | TN97-08 | KC999968.1 | OX1 |  | wzx/wzy | 99% |
| *A. hydrophila* | MN98-04 | KC999969.1 | OX8 |  | wzm/wzt |  |
| *A. hydrophila* | AL97-91 | KC999966.1 | OX8 |  | wzm/wzt | 100% |
| *A. hydrophila* | AL06-01 | KC999970.1 | OX9 |  | wzm/wzt |  |
| *A. hydrophila* | AH-3 | EU274663.1 | O34 |  | wzx/wzy |  |
