# Supplementary material for: Identifying genetic diversity of O antigens in Aeromonas hydrophila for molecular serotype detection
Source: PLoS One. 2018 Sep 5;13(9):e0203445. doi: 10.1371/journal.pone.0203445 (PMC6124807; doi:10.1371/journal.pone.0203445)
Supplement: S5 Table — (DOC) [file pone.0203445.s005.doc]

**S5 Table. Characteristics of the ORFs in 14 newly OGCs.**

***Aeromonas hydrophila* O7**

| **Orf** | **Gene**  **name** | **Location** | **Strand** | **GC**  **Content%** | **Similar protein, strains, Genbank accession number** | **%Identical/**  **%Similar** | **Putative functions** |
| --- | --- | --- | --- | --- | --- | --- | --- |
| 1 | acrB | 1..3150 | + | 60.86 | AcrB protein [Aeromonas hydrophila TN97-08] AID71002.1 | 98.30/95.30 | Multidrug efflux pump subunit AcrB |
| 2 | rmlB | 3731..4816 | + | 56.63 | dTDP-glucose 4,6-dehydratase [Aeromonas veronii] WP_076492308.1 | 99.20/98.10 | dTDP-glucose 4,6-dehydratase |
| 3 | rmlD | 4816..5703 | + | 60.59 | dTDP-4-dehydrorhamnose reductase [Aeromonas caviae] WP_069785289.1 | 99.70/98.30 | dTDP-4-dehydrorhamnose reductase |
| 4 | rmlA | 5816..6694 | + | 52.45 | glucose-1-phosphate thymidylyltransferase RfbA [Aeromonas hydrophila] WP_101149011.1 | 98.30/96.20 | glucose-1-phosphate thymidylyltransferase |
| 5 | rmlC | 6757..7311 | + | 42.34 | dTDP-4-dehydrorhamnose 3,5-epimerase [Aeromonas veronii]ATY78208.1 dTDP-4-dehydrorhamnose 3,5-epimerase [Aeromonas veronii] WP_100654181.1 | 99.50/98.90 | dTDP-4-dehydrorhamnose 3,5-epimerase |
| 6 | wzm | 7314..8132 | + | 33.58 | sugar ABC transporter permease [Aeromonas veronii] WP_100654180.1 | 100.00/100.00 | ABC transporter permease |
| 7 | wzt | 8122..9363 | + | 37.36 | ABC transporter ATP-binding protein [Aeromonas veronii] WP_100654179.1 | 100.00/100.00 | ABC transporter ATP-binding protein |
| 8 | wbxZ | 9369..12218 | + | 37.23 | hypothetical protein [Aeromonas veronii] WP_100654178.1 | 99.80/99.50 | glycosyltransferase |
| 9 | mnaA | 12232..13341 | + | 42.79 | UDP-N-acetylglucosamine 2-epimerase [Aeromonas veronii] WP_100654177.1 | 100.00/99.70 | UDP-N-acetylglucosamine 2-epimerase |
| 10 | wbxX | 13338..14423 | + | 39.04 | glycosyltransferase [Aeromonas hydrophila] AID71050.1 | 90.30/81.70 | Glycosyl transferase |
| 11 | wbxW | 14414..15223 | + | 43.27 | glycosyl transferase [Aeromonas veronii] WP_100654175.1 | 99.30/97.00 | Glycosyl transferase |
| 12 | galE | 15223..16179 | + | 52.77 | NAD-dependent dehydratase [Aeromonas veronii] WP_031227811.1 | 98.10/97.80 | UDP-glucose-4-epimerase |
| 13 | wbpI | 16179..17204 | + | 54.78 | glycosyl transferase [Aeromonas veronii] WP_042080970.1 | 98.80/98.80 | glycosyltransferase |
| 14 | wbgZ | 17201..19186 | + | 53.98 | nucleoside-diphosphate sugar epimerase [Aeromonas veronii] WP_042080969.1 | 99.80/99.70 | nucleoside-diphosphate sugar epimerase |
| 15 | wecA | 19667..20731 | + | 51.64 | undecaprenyl-phosphate alpha-N-acetylglucosaminyl 1-phosphate transferase [Aeromonas veronii] WP_100654171.1 | 98.00/96.90 | undecaprenyl-phosphate alpha-N-acetylglucosaminyl 1-phosphate transferase |
| 16 | orf1 | 20840..21862 | + | 38.22 | hypothetical protein [Vibrio cholerae] WP_000734260.1 | 72.80/59.60 | Hypothetical protein |
| 17 | csaB | 21884..23098 | + | 37.40 | polysaccharide biosynthesis protein [Aeromonas hydrophila] WP_011706687.1 | 93.10/86.40 | polysaccharide biosynthesis protein |
| 18 | wbxV | 23155..24249 | + | 35.53 | glycoside hydrolase [Aeromonas hydrophila] KGY52520.1 | 97.30/91.80 | glycoside hydrolase |
| 19 | wbxJ | 24253..25074 | + | 35.28 | glycosyl transferase [Aeromonas hydrophila] WP_011706685.1 | 94.50/86.80 | glycoside hydrolase |
| 20 | GT1 | 25076..25870 | + | 37.23 | glycosyl transferase family protein [Aeromonas hydrophila] AID71039.1 | 94.30/87.90 | glycosyltransferase |
| 21 | orf2 | 25971..27125 | + | 30.13 | O-antigen polymerase [Aeromonas hydrophila] AID71038.1 | 94.00/84.40 | Hypothetical protein |
| 22 | wcaG | 27122..28135 | + | 48.03 | protein CapI [Aeromonas salmonicida] WP_059168593.1 | 99.10/99.10 | protein CapI |
| 23 | wbpO | 28148..29314 | + | 49.61 | nucleotide sugar dehydrogenase [Aeromonas veronii] WP_100654161.1 | 100.00/99.50 | UDP-glucose 6-dehydrogenase |
| 24 | wza | 29636..30751 | + | 58.78 | polysaccharide export protein Wza [Aeromonas veronii] WP_100654159.1 | 100.00/99.50 | polysaccharide export protein |
| 25 | wzb | 30987..31415 | + | 58.74 | protein-tyrosine-phosphatase [Aeromonas sobria] WP_042018640.1 | 100.00/100.00 | protein-tyrosine-phosphatase |
| 26 | wzc | 31478..33652 | + | 55.91 | tyrosine-protein kinase [Aeromonas sp. DNP9] WP_069554775.1 | 99.70/99.30 | tyrosine-protein kinase |
| 27 | orf3 | 33933..34193 | + | 33.97 | hypothetical protein [Aeromonas sp. DNP9] WP_069554774.1 | 98.80/97.70 | Hypothetical protein |
| 28 | ymcC | 34251..34895 | + | 55.94 | YjbF family lipoprotein [Aeromonas veronii] WP_100654156.1 | 99.10/99.10 | YjbF family lipoprotein |
| 29 | orf4 | 34892..35638 | + | 50.39 | hypothetical protein [Aeromonas veronii] WP_042080341.1 | 99.60/98.00 | Hypothetical protein |
| 30 | orf5 | 35758..37749 | + | 51.85 | YjbH domain-containing protein [Aeromonas veronii] WP_100654154.1 | 99.80/99.20 | Hypothetical protein |
| 31 | waaL | 37823..39583 | + | 49.16 | ligase [Aeromonas veronii]ATY79321.1 ligase [Aeromonas veronii] WP_100654834.1 | 99.10/99.00 | ligase |
| 32 | hns | 40141..40551 | + | 53.53 | Histone-like nucleoid structuring protein [Aeromonas veronii B565] AEB50806.1 | 100.00/100.00 | Histone-like nucleoid structuring protein |
| 33 | oprM | 41088..42452 | - | 62.27 | Outer membrane protein OprM[Aeromonas dhakensis] WP_010633426.1 | 99.10/98.70 | Outer membrane protein OprM |

***Aeromonas hydrophila*** O9

| **Orf** | **Gene**  **name** | **Location** | **Strand** | **GC**  **Content%** | **Similar protein, strains, Genbank accession number** | **%Identical/**  **%Similar** | **Putative functions** |
| --- | --- | --- | --- | --- | --- | --- | --- |
| 1 | oprM | 1..1365 | + | 62.49 | transporter [Aeromonas enteropelogenes] WP_026456497.1 | 98.90/98.20 | Outer membrane protein OprM |
| 2 | orf2 | 1776..2183 | - | 52.70 | transcriptional regulator [Aeromonas enteropelogenes] WP_061475545.1 | 100.00/100.00 | DNA-binding protein H-NS |
| 3 | waaL | 2791..4524 | - | 49.37 | ligase [Aeromonas enteropelogenes] WP_042072399.1 | 99.60/99.50 | ligase |
| 4 | wzz | 4650..5720 | - | 54.06 | hypothetical protein [Aeromonas fluvialis] WP_042009712.1 | 83.10/67.30 | O-antigen chain length determinant protein |
| 5 | rmlC | 5713..6273 | - | 53.65 | dTDP-4-dehydrorhamnose 3,5-epimerase [Aeromonas fluvialis] WP_042009714.1 | 80.80/70.10 | dTDP-4-dehydrorhamnose 3,5-epimerase |
| 6 | rmlA2 | 6303..7226 | - | 53.36 | glucose-1-phosphate thymidylyltransferase [Aeromonas enteropelogenes] WP_061475546.1 | 98.00/97.40 | Glucose-1-phosphate thymidylyltransferase |
| 7 | rmlD | 7226..8110 | - | 53.56 | hypothetical protein [Aeromonas enteropelogenes] WP_061475547.1 | 98.60/95.90 | dTDP-4-dehydrorhamnose reductase |
| 8 | GT1 | 8097..9158 | - | 44.82 | undecaprenyl-phosphate alpha-N-acetylglucosaminyl 1-phosphate transferase [Aeromonas enteropelogenes] WP_061475548.1 | 98.30/97.20 | Undecaprenyl-phosphate alpha-N-acetylglucosaminyl 1-phosphate transferase |
| 9 | manB | 9214..10638 | - | 47.58 | phosphomannomutase [Aeromonas enteropelogenes WP_061475549.1 | 97.00/94.90 | phosphomannomutase |
| 10 | GT2 | 10631..11386 | - | 40.08 | glycosyltransferase [Aeromonas sobria] WP_101347526.1 | 98.80/95.20 | glycosyltransferase |
| 11 | manC | 11386..12792 | - | 52.03 | mannose-1-phosphate guanylyltransferase/mannose-6-phosphate isomerase [Aeromonas sobria] WP_101347527.1 | 98.30/97.20 | Mannose-1-phosphate guanylyltransferase |
| 12 | gmm | 12806..13162 | - | 48.47 | GDP-mannose mannosyl hydrolase [Aeromonas enteropelogenes] WP_061475551.1 | 89.80/84.70 | GDP-mannose mannosyl hydrolase |
| 13 | fcl | 13278..14258 | - | 51.58 | GDP-fucose synthetase [Aeromonas hydrophila] WP_043160810.1 | 96.00/92.00 | GDP-L-fucose synthase |
| 14 | gmd | 14262..15368 | - | 47.25 | GDP-mannose 4,6-dehydratase [Aeromonas hydrophila] WP_043119174.1 | 98.10/96.50 | GDP-mannose 4,6-dehydratase |
| 15 | GT3 | 15390..16628 | - | 34.06 | group 1 glycosyl transferase [Aeromonas hydrophila] AHW40518.1 | 88.20/75.20 | glycosyltransferase |
| 16 | GT4 | 16625..17674 | - | 33.43 | glycosyl transferase, group 1 family protein [Aeromonas hydrophila] AID70991.1 | 85.60/70.10 | glycosyltransferase |
| 17 | GT5 | 17677..18858 | - | 37.56 | glycosyl transferase group 1 [Aeromonas hydrophila] AHW40520.1 | 83.90/72.60 | glycosyltransferase |
| 18 | wzy | 19327..20544 | - | 32.92 | hypothetical protein [Pectobacterium carotovorum] WP_015839582.1 | 52.80/34.60 | O antigen polymerase |
| 19 | wvaA | 20551..21408 | - | 31.35 | glycosyl transferase family 2 [Pectobacterium carotovorum] WP_010296778.1 | 78.30/62.30 | glycosyltransferase |
| 20 | wzx | 21435..22697 | - | 35.55 | O-antigen flippase [Aeromonas sp. YN13HZO-058] WP_075113079.1 | 82.70/67.80 | O antigen flippase |
| 21 | fdtB | 22694..23797 | - | 36.96 | aminotransferase class V-fold PLP-dependent enzyme [Aeromonas caviae] WP_082189314.1 | 86.90/77.90 | dTDP-6-deoxy-D-xylo-hex-3-ulose aminase |
| 22 | qdtF | 23857..25059 | - | 32.42 | formyl transferase [Vibrio vulnificus]ARN64690.1 Methionyl-tRNA formyltransferase [Vibrio vulnificus] WP_085760666.1 | 74.90/55.20 | dTDP-D-Fuc3N acetylase |
| 23 | fdtA | 25049..25456 | - | 36.28 | dTDP-6-deoxy-3,4-keto-hexulose isomerase [Aeromonas sp. 62-46] OJW62522.1 | 86.40/72.00 | dTDP-6-deoxy-hex-4-ulose isomerase |
| 24 | rmlA | 25453..26325 | - | 41.58 | glucose-1-phosphate thymidylyltransferase [Aeromonas finlandiensis] WP_033137071.1 | 95.50/90.30 | Glucose-1-phosphate thymidylyltransferase |
| 25 | rmlB | 26325..27410 | - | 53.47 | dTDP-glucose 4,6-dehydratase [Aeromonas veronii] WP_043818674.1 | 96.70/92.80 | dTDP-glucose 4,6-dehydratase |
| 26 | acrB | 28089..31238 | - | 60.41 | multidrug efflux RND transporter permease subunit [Aeromonas enteropelogenes] WP_042072114.1 | 100.00/100.00 | Multidrug efflux pump subunit AcrB |

***Aeromonas hydrophila*** O10

| **Orf** | **Gene**  **name** | **Location** | **Strand** | **GC**  **Content%** | **Similar protein, strains, Genbank accession number** | **%Identical/**  **%Similar** | **Putative functions** |
| --- | --- | --- | --- | --- | --- | --- | --- |
| 1 | oprM | 1..1362 | + | 60.87 | transporter [Aeromonas lacus] WP_033113481.1 | 99.30/98.90 | Outer membrane protein OprM |
| 2 | hns | 1757..2167 | - | 53.04 | transcriptional regulator [Aeromonas] WP_033113480.1 | 100.00/99.30 | DNA-binding protein H-NS |
| 3 | waaL | 2707..4449 | - | 49.11 | ligase [Aeromonas lacus] WP_033113479.1 | 95.20/92.80 | ligase |
| 4 | wecA | 4743..6014 | - | 50.16 | glycosyl transferase [Aeromonas caviae] WP_042017510.1 | 95.30/89.80 | UDP-N-acetylgalactosamine-undecaprenyl-phosphate N-acetylgalactosaminephosphotransferase |
| 5 | GT1 | 6058..7215 | - | 41.97 | glycosyl transferase [Aeromonas caviae] KGY67619.1 | 85.40/74.90 | glycosyltransferase |
| 6 | GT2 | 7271..8356 | - | 45.95 | glycosyl transferase [Aeromonas salmonicida] WP_058394230.1 | 83.40/73.10 | glycosyltransferase |
| 7 | GT3 | 8371..9345 | - | 32.92 | hypothetical protein [Escherichia coli] WP_072731165.1 | 61.00/41.60 | glycosyltransferase |
| 8 | orf1 | 9419..10387 | - | 32.51 | hypothetical protein [Pectobacterium carotovorum] WP_039513396.1 | 66.00/48.30 | hypothetical protein |
| 9 | glf | 10384..11514 | - | 39.43 | UDP-galactopyranose mutase [Aeromonas salmonicida] WP_058394232.1 | 94.50/87.80 | UDP-galactopyranose mutase |
| 10 | orf2 | 11504..12763 | - | 38.81 | hypothetical protein [Aeromonas salmonicida] WP_058394233.1 | 91.70/86.10 | hypothetical protein |
| 11 | rmlC | 12766..13317 | - | 45.47 | dTDP-4-dehydrorhamnose 3,5-epimerase [Aeromonas salmonicida] WP_059168599.1 | 96.20/91.80 | dTDP-4-dehydrorhamnose 3,5-epimerase |
| 12 | rmlA | 13379..14257 | - | 53.13 | glucose-1-phosphate thymidylyltransferase RfbA [Aeromonas hydrophila] WP_101149011.1 | 97.90/97.60 | Glucose-1-phosphate thymidylyltransferase |
| 13 | rmlD | 14374..15255 | - | 59.07 | dTDP-4-dehydrorhamnose reductase [Aeromonas sp. CU5] WP_098969878.1 | 99.00/98.30 | dTDP-4-dehydrorhamnose reductase |
| 14 | rmlB | 15255..16361 | - | 56.73 | dTDP-glucose 4,6-dehydratase [Aeromonas jandaei] WP_042032474.1 | 98.60/97.30 | dTDP-glucose 4,6-dehydratase |
| 15 | wbgZ | 17006..18991 | - | 51.31 | nucleoside-diphosphate sugar epimerase [Aeromonas veronii] WP_100654172.1 | 98.30/96.50 | UDP-N-acetyl-alpha-D-glucosamine C6 dehydratase |
| 16 | GT4 | 18988..19866 | - | 54.00 | glycosyl transferase [Aeromonas sp. DNP9] WP_069554782.1 | 99.70/99.00 | glycosyltransferase |
| 17 | galE | 20013..20978 | - | 54.04 | NAD-dependent dehydratase [Aeromonas hydrophila] WP_043158922.1 | 98.40/96.80 | N-acetyl-alpha-D-glucosaminyl-diphospho-ditrans,octacis-undecaprenol 4-epimerase |
| 18 | GT5 | 20975..22099 | - | 44.00 | glycosyl transferase family 1 [Aeromonas dhakensis] WP_095592375.1 | 82.50/68.20 | glycosyltransferase |
| 19 | GT6 | 22099..23241 | - | 46.19 | mannosyltransferase [Dickeya zeae] WP_038907078.1 | 82.60/69.70 | glycosyltransferase |
| 20 | orf3 | 23238..24128 | - | 45.90 | GDP-6-deoxy-D-lyxo-4-hexulose reductase [Dickeya zeae] WP_038903353.1 | 85.00/74.10 | hypothetical protein |
| 21 | gmd | 24180..25214 | - | 45.60 | GDP-mannose 4,6-dehydratase [Pantoea agglomerans] WP_031591340.1 | 95.90/90.40 | GDP-mannose 4,6-dehydratase |
| 22 | GT7 | 25235..25987 | - | 36.39 | glycosyl transferase family 2 [Vibrio tasmaniensis] WP_065106114.1 | 88.80/77.70 | glycosyltransferase |
| 23 | GT8 | 26309..27397 | - | 37.31 | hypothetical protein [Vibrio tasmaniensis] WP_065106112.1 | 85.80/71.00 | glycosyltransferase |
| 24 | wesG | 27390..28040 | - | 32.87 | HAD family phosphatase [Vibrio tasmaniensis] WP_065106111.1 | 91.50/84.90 | Phosphorylated carbohydrates phosphatase |
| 25 | wesF | 28021..28656 | - | 35.33 | hypothetical protein [Vibrio tasmaniensis] WP_065106110.1 | 72.40/55.20 | hypothetical protein |
| 26 | GT9 | 28665..29543 | - | 34.59 | hypothetical protein [Yersinia frederiksenii] WP_050106846.1 | 64.20/45.00 | glycosyltransferase |
| 27 | GT10 | 29587..30732 | - | 32.77 | glycosyltransferase family 1 protein [Dickeya zeae] WP_080649052.1 | 59.10/41.70 | glycosyltransferase |
| 28 | orf4 | 30701..31918 | - | 32.46 | hypothetical protein [Yersinia frederiksenii] WP_050106848.1 | 64.10/48.30 | hypothetical protein |
| 29 | wzt | 31918..33129 | - | 31.45 | hypothetical protein [Dickeya zeae] WP_038903342.1 | 68.50/54.10 | ABC transporter ATP-binding protein |
| 30 | wzm | 33129..33926 | - | 34.90 | ABC transporter [Vibrio cholerae] WP_032473383.1 | 88.50/70.50 | ABC transporter permease |
| 31 | manB | 33931..35292 | - | 44.74 | phosphomannomutase [Aeromonas] WP_080760782.1 | 85.80/75.70 | Phosphomannomutase/phosphoglucomutase |
| 32 | manC | 35314..36717 | - | 48.09 | mannose-1-phosphate guanylyltransferase/mannose-6-phosphate isomerase [Aeromonas hydrophila] WP_011706705.1 | 91.80/82.40 | Mannose-1-phosphate guanylyltransferase RfbM |
| 33 | orf5 | 36790..36948 | - | 41.45 | hypothetical protein | | hypothetical protein |
| 34 | rmlC1 | 37010..37303 | - | 46.94 | dTDP-4-dehydrorhamnose 3,5-epimerase [Aeromonas australiensis] WP_040094730.1 | 94.80/90.60 | dTDP-4-dehydrorhamnose 3,5-epimerase |
| 35 | rmlA2 | 37364..37636 | - | 50.18 | glucose-1-phosphate thymidylyltransferase RfbA [Aeromonas hydrophila] WP_101149011.1 | 97.80/93.30 | Glucose-1-phosphate thymidylyltransferase |
| 36 | rmlA3 | 37672..38109 | - | 52.51 | glucose-1-phosphate thymidylyltransferase [Aeromonas salmonicida] WP_059168600.1 | 95.20/93.80 | Glucose-1-phosphate thymidylyltransferase 1 |
| 37 | acrB | 39108..42257 | - | 61.62 | multidrug efflux RND transporter permease subunit [Aeromonas jandaei] WP_042032472.1 | 100.00/99.80 | Multidrug efflux pump subunit AcrB |

***Aeromonas hydrophila*** O13

| **Orf** | **Gene**  **name** | **Location** | **Strand** | **GC**  **content** | **Similar protein, strains, Genbank accession number** | **%Identical/**  **%Similar** | **Putative functions** |
| --- | --- | --- | --- | --- | --- | --- | --- |
| 1 | acrB | 1..3150 | + | 62.92 | multidrug efflux RND transporter permease subunit [Aeromonas encheleia] WP_042654988.1 | 98.00/95.00 | Multidrug efflux pump subunit AcrB |
| 2 | oprM | 3143..4552 | + | 64.82 | multidrug transporter [Aeromonas hydrophila] WP_017786283.1 | 96.20/92.80 | Outer membrane protein OprM |
| 3 | rmlB | 5332..6417 | + | 57.74 | dTDP-glucose 4,6-dehydratase [Aeromonas hydrophila] WP_024942568.1 | 99.40/99.20 | dTDP-glucose 4,6-dehydratase |
| 4 | rmlD | 6417..7304 | + | 60.92 | NAD(P)-dependent oxidoreductase [Aeromonas hydrophila] WP_024942569.1 | 100.00/100.00 | dTDP-4-dehydrorhamnose reductase |
| 5 | rmlA | 7417..8295 | + | 50.85 | glucose-1-phosphate thymidylyltransferase [Aeromonas hydrophila] WP_041217355.1 | 99.00/98.30 | Glucose-1-phosphate thymidylyltransferase 1 |
| 6 | rmlC | 8358..8897 | + | 40.74 | dTDP-4-dehydrorhamnose 3,5-epimerase [Aeromonas hydrophila] WP_101613939.1 | 99.00/96.00 | dTDP-4-dehydrorhamnose 3,5-epimerase |
| 7 | wzm | 8899..9708 | + | 30.62 | ABC transporter permease [Aeromonas hydrophila] WP_080689618.1 | 100.00/100.00 | ABC transporter permease |
| 8 | wzt | 9698..11002 | + | 35.17 | ABC transporter ATP-binding protein [Aeromonas hydrophila] WP_080689623.1 | 100.00/100.00 | ABC transporter ATP-binding protein |
| 9 | GT1 | 11015..13717 | + | 33.37 | hypothetical protein [Aeromonas hydrophila] WP_080689617.1 | 100.00/99.90 | glycosyltransferase |
| 10 | GT2 | 13704..14780 | + | 34.08 | hypothetical protein [Aeromonas hydrophila] WP_080689616.1 | 100.00/100.00 | glycosyltransferase |
| 11 | mnaA | 14792..15901 | + | 43.69 | UDP-N-acetylglucosamine 2-epimerase (non-hydrolyzing) [Aeromonas hydrophila] WP_080756313.1 | 100.00/99.70 | UDP-N-acetylglucosamine-2-epimerase |
| 12 | GT3 | 15898..16905 | + | 38.69 | hypothetical protein [Aeromonas hydrophila] WP_080689614.1 | 99.40/99.10 | glycosyltransferase |
| 13 | GT4 | 16975..17784 | + | 42.26 | glycosyltransferase family 2 protein [Aeromonas hydrophila] WP_080689613.1 | 93.30/89.60 | glycosyltransferase |
| 14 | galE | 17784..18740 | + | 53.92 | NAD-dependent dehydratase [Aeromonas veronii] WP_076492336.1 | 96.20/93.70 | N-acetyl-alpha-D-glucosaminyl-diphospho-ditrans,octacis-undecaprenol 4-epimerase |
| 15 | GT5 | 18740..19765 | + | 50.88 | glycosyl transferase [Aeromonas sp. DNP9] WP_069554782.1 | 95.30/93.50 | glycosyltransferase |
| 16 | wbgZ | 19762..21747 | + | 52.72 | nucleoside-diphosphate sugar epimerase WP_024942476.1 | 98.50/97.90 | UDP-N-acetyl-alpha-D-glucosamine C6 dehydratase |
| 17 | wecA | 22232..23302 | + | 47.41 | undecaprenyl-phosphate alpha-N-acetylglucosaminyl 1-phosphate transferase [Aeromonas hydrophila] WP_024942475.1 | 100.00/99.70 | Undecaprenyl-phosphate alpha-N-acetylglucosaminyl 1-phosphate transferase |
| 18 | rimK | 23484..24386 | + | 50.89 | hypothetical protein [Aeromonas hydrophila] WP_080689612.1 | 100.00/100.00 | hypothetical protein |
| 19 | adl | 24379..25494 | + | 38.61 | hypothetical protein [Aeromonas hydrophila] WP_080689611.1 | 97.30/94.10 | Alanine dehydrogenase |
| 20 | csaB | 25496..26716 | + | 40.05 | polysaccharide biosynthesis protein [Aeromonas hydrophila] WP_011706687.1 | 86.90/72.00 | transporter |
| 21 | orf1 | 26771..27877 | + | 35.87 | hypothetical protein [Xenorhabdus bovienii] WP_038197792.1 | 49.50/34.20 | hypothetical protein |
| 22 | GT6 | 27886..28995 | + | 29.65 | hypothetical protein PO25_01755 [Vibrio anguillarum] AQM28719.1 | 82.10/64.40 | glycosyltransferase |
| 23 | GT7 | 29011..29832 | + | 37.39 | glycosyl transferase [Vibrio anguillarum] AQM28720.1 | 80.60/68.10 | glycosyltransferase |
| 24 | wcaG | 29925..31022 | + | 37.11 | protein CapI [Aeromonas salmonicida] WP_059168593.1 | 98.80/97.90 | UDP-N-acetylglucosamine 4-epimerase |
| 25 | ugd | 31038..32204 | + | 46.19 | UDP-glucose 6-dehydrogenase [Aeromonas hydrophila] WP_024942468.1 | 99.20/98.50 | UDP-glucose 6-dehydrogenase |
| 26 | wza | 32537..33652 | + | 57.62 | polysaccharide export protein Wza [Aeromonas hydrophila] WP_080689607.1 | 100.00/100.00 | polysaccharide export protein |
| 27 | wzb | 33837..34265 | + | 57.81 | protein-tyrosine-phosphatase [Aeromonas hydrophila] WP_024942466.1 | 100.00/100.00 | protein-tyrosine-phosphatase |
| 28 | wzc | 34328..36502 | + | 54.21 | tyrosine protein kinase [Aeromonas] WP_043169738.1 | 99.70/99.40 | Tyrosine-protein kinase wzc |
| 29 | ymcC | 36949..37638 | + | 54.62 | YjbF family lipoprotein [Aeromonas dhakensis] WP_095592389.1 | 98.70/98.30 | YjbF family lipoprotein |
| 30 | orf2 | 37635..38393 | + | 44.78 | hypothetical protein [Aeromonas] WP_080760770.1 | 96.80/94.00 | hypothetical protein |
| 31 | orf3 | 38393..40474 | + | 48.01 | membrane protein [Aeromonas] WP_043169736.1 | 99.60/98.70 | hypothetical protein |
| 32 | waaL | 40567..42297 | + | 62.68 | ligase [Aeromonas dhakensis] WP_095592390.1 | 97.90/97.00 | ligase |

***Aeromonas hydrophila*** O16

| **Orf** | **Gene**  **name** | **Location** | **Strand** | **GC**  **Content%** | **Similar protein, strains, Genbank accession number** | **%Identical/**  **%Similar** | **Putative functions** |
| --- | --- | --- | --- | --- | --- | --- | --- |
| 1 | acrB | 1..2505 | + | 63.19 | multidrug efflux RND transporter permease subunit [Aeromonas dhakensis] WP_042051937.1 | 100.00/99.70 | Multidrug efflux pump subunit AcrB |
| 2 | orf1 | 2462..3145 | + | 63.35 | AcrB/AcrD/AcrF family protein [Aeromonas hydrophila] WP_101149015.1 | 100.00/100.00 | hypothetical protein |
| 3 | oprM | 3138..4547 | + | 64.97 | outer membrane protein OprM [Aeromonas dhakensis] WP_010633426.1 | 99.60/99.40 | Outer membrane protein OprM |
| 4 | rmlB | 5316..6401 | + | 56.91 | dTDP-glucose 4,6-dehydratase [Aeromonas hydrophila] WP_044801222.1 | 98.60/98.60 | dTDP-glucose 4,6-dehydratase |
| 5 | rmlD | 6401..7288 | + | 61.26 | NAD(P)-dependent oxidoreductase [Aeromonas veronii] WP_040065956.1 | 99.00/98.00 | dTDP-4-dehydrorhamnose reductase |
| 6 | rmlA | 7402..8280 | + | 51.76 | glucose-1-phosphate thymidylyltransferase [Aeromonas dhakensis] WP_042051943.1 | 98.60/98.60 | Glucose-1-phosphate thymidylyltransferase 1 |
| 7 | rmlC | 8286..8843 | + | 39.79 | dTDP-4-dehydrorhamnose 3,5-epimerase [Aeromonas dhakensis] WP_082038655.1 | 100.00/99.50 | dTDP-4-dehydrorhamnose 3,5-epimerase |
| 8 | fdtA | 8834..9232 | + | 42.36 | dTDP-6-deoxy-3,4-keto-hexulose isomerase [Aeromonas caviae] KEP88971.1 | 95.00/91.00 | dTDP-6-deoxy-D-xylo-hex-3-ulose aminase |
| 9 | fdtC | 9229..9978 | + | 39.33 | acetyltransferase [Aeromonas dhakensis] WP_042051948.1 | 100.00/100.00 | dTDP-D-Fuc3N acetylase |
| 10 | fdtB | 10384..11487 | + | 41.94 | aminotransferase [Aeromonas dhakensis] WP_042051950.1 | 100.00/100.00 | dTDP-3-amino-3,6-dideoxy-alpha-D-galactopyranose transaminase |
| 11 | wzx | 11484..12734 | + | 40.29 | WzxB protein [Aeromonas dhakensis] WP_042051951.1 | 99.80/99.80 | O antigen flippase |
| 12 | GT1 | 12744..14168 | + | 34.39 | glycosyl transferase [Citrobacter freundii] WP_003842211.1 | 61.60/45.40 | glycosyltransferase |
| 13 | wzy | 14212..15525 | + | 32.04 | membrane protein [Escherichia coli] WP_001484688.1 | 68.20/51.30 | O antigen polymerase |
| 14 | GT2 | 15522..16361 | + | 36.79 | hypothetical protein [Aeromonas dhakensis] WP_042051953.1 | 99.60/98.60 | glycosyltransferase |
| 15 | GT3 | 16424..17233 | + | 33.46 | amylovoran biosynthesis protein AmsE [Aeromonas dhakensis] WP_082038656.1 | 100.00/99.60 | glycosyltransferase |
| 16 | GT4 | 17261..18541 | + | 40.20 | sugar transferase [Aeromonas dhakensis] WP_082038668.1 | 100.00/99.10 | UDP-N-acetylgalactosamine-undecaprenyl-phosphate N-acetylgalactosaminephosphotransferase |
| 17 | wzz | 18625..19689 | + | 40.19 | hypothetical protein [Aeromonas dhakensis] WP_082038657.1 | 100.00/99.40 | O-antigen chain length determinant protein |
| 18 | waaL | 19766..21484 | + | 62.71 | hypothetical protein [Aeromonas hydrophila] AAM74488.1 | 98.20/97.70 | ligase |

***Aeromonas hydrophila*** O19

| **Orf** | **Gene**  **name** | **Location** | **Strand** | **GC**  **Content%** | **Similar protein, strains, Genbank accession number** | **%Identical/**  **%Similar** | **Putative functions** |
| --- | --- | --- | --- | --- | --- | --- | --- |
| 1 | acrB | 1..3150 | + | 60.41 | multidrug transporter [Aeromonas veronii] WP_058059856.1 | 99.80/99.70 | Multidrug efflux pump subunit AcrB |
| 2 | orf1 | 3831..3968 | - | 43.29 | No hits found | / |  |
| 3 | wecB | 4143..5267 | + | 41.23 | UDP-N-acetyl glucosamine 2-epimerase [Aeromonas sobria] WP_101315516.1 | 96.80/95.70 | UDP-N-acetylglucosamine 2-epimerase |
| 4 | mnaB | 5324..6589 | + | 29.88 | UDP-N-acetyl-D-mannosamine dehydrogenase [Aeromonas sobria] WP_101315514.1 | 97.40/95.50 | UDP-N-acetyl-D-mannosamine dehydrogenase |
| 5 | wzx | 6586..7824 | + | 36.25 | hypothetical protein [Aeromonas sobria] WP_101315512.1 | 95.00/85.60 | O antigen flippase |
| 6 | wzy | 8284..9483 | + | 29.67 | hypothetical protein AOX56_01425 [Aeromonas sobria] PKQ83206.1 | 94.20/89.50 | O antigen polymerase |
| 7 | wvcJ | 9471..11366 | + | 33.91 | asparagine synthase (glutamine-hydrolyzing) [Aeromonas sobria] WP_101315506.1 | 97.00/93.00 | Asparagine synthetase |
| 8 | orf4 | 11359..13248 | + | 32.65 | hypothetical protein [Aeromonas sobria] WP_101315504.1 | 94.80/89.70 | hypothetical protein |
| 9 | GT1 | 13245..14489 | + | 43.13 | glycosyltransferase WbuB [Aeromonas sobria] WP_101315502.1 | 96.60/95.60 | glycosyltransferase |
| 10 | wecA | 14473..15069 | + | 41.71 | sugar transferase [Aeromonas sobria] WP_101315500.1 | 95.90/94.40 | UDP-N-acetylgalactosamine-undecaprenyl-phosphate N-acetylgalactosaminephosphotransferase |
| 11 | fdtC | 15059..15703 | + | 43.10 | acetyltransferase [Aeromonas sobria] WP_101315571.1 | 92.80/86.50 | dTDP-D-Fuc3N acetylase |
| 12 | fdtB | 15732..16907 | + | 47.19 | DegT/DnrJ/EryC1/StrS aminotransferase family protein [Aeromonas sobria] WP_101315569.1 | 98.50/95.70 | dTDP-3-amino-3,6-dideoxy-alpha-D-galactopyranose transaminase |
| 13 | wbgZ | 16968..18929 | + | 49.08 | PII uridylyl-transferase [Aeromonas] WP_019445293.1 | 100.00/99.80 | UDP-N-acetyl-alpha-D-glucosamine C6 dehydratase |
| 14 | rmlB | 19129..20217 | + | 56.75 | dTDP-glucose 4,6-dehydratase [Aeromonas] WP_019445292.1 | 99.70/99.70 | dTDP-glucose 4,6-dehydratase |
| 15 | rmlD | 20217..21104 | + | 60.36 | dTDP-4-dehydrorhamnose reductase [Aeromonas veronii] WP_058057705.1 | 99.70/99.70 | dTDP-4-dehydrorhamnose reductase |
| 16 | rmlA | 21219..22106 | + | 54.17 | glucose-1-phosphate thymidylyltransferase [Aeromonas sp. 159] WP_019445290.1 | 99.00/98.00 | Glucose-1-phosphate thymidylyltransferase 1 |
| 17 | rmlC | 22190..22747 | + | 46.24 | dTDP-4-dehydrorhamnose 3,5-epimerase [Aeromonas sp. ZOR0001] WP_081990737.1 | 93.90/93.90 | dTDP-4-dehydrorhamnose 3,5-epimerase |
| 18 | wzz | 22801..23859 | + | 48.91 | hypothetical protein [Aeromonas australiensis] WP_040094846.1 | 94.00/90.60 | O-antigen chain length determinant protein |
| 19 | waaL | 24731..26461 | + | 49.68 | ligase [Aeromonas sp. ZOR0001] WP_047439131.1 | 97.20/95.50 | ligase |
| 20 | hns | 27068..27478 | + | 52.80 | transcriptional regulator [Aeromonas] WP_040065985.1 | 100.00/100.00 | DNA-binding protein H-NS |
| 21 | oprM | 28014..29378 | - | 61.69 | outer membrane efflux protein [Aeromonas hydrophila ML09-119] AGM44997.1 | 88.30/72.00 | Outer membrane protein OprM |

***Aeromonas hydrophila*** O23

| **Orf** | **Gene**  **name** | **Location** | **Strand** | **GC**  **Content%** | **Similar protein, strains, Genbank accession number** | **%Identical/**  **%Similar** | **Putative functions** |
| --- | --- | --- | --- | --- | --- | --- | --- |
| 1 | oprM | 1..1365 | + | 61.69 | transporter [Aeromonas] WP_069527417.1 | 98.50/97.60 | Outer membrane protein OprM |
| 2 | hns | 1902..2312 | - | 53.04 | DNA-binding protein [Aeromonas] WP_005344269.1 | 100.00/100.00 | DNA-binding protein H-NS |
| 3 | waaL | 2870..4630 | - | 49.34 | ligase [Aeromonas veronii]ATY79321.1 ligase [Aeromonas veronii] WP_100654834.1 | 99.30/99.10 | ligase |
| 4 | orf2 | 4704..6815 | - | 51.89 | membrane protein [Aeromonas veronii] WP_042080340.1 | 99.60/99.60 | hypothetical protein |
| 5 | orf3 | 6815..7561 | - | 55.96 | hypothetical protein [Aeromonas veronii] WP_042080341.1 | 98.00/96.80 | hypothetical protein |
| 6 | ymcC | 7558..8202 | - | 50.54 | YjbF family lipoprotein [Aeromonas veronii]ATY78182.1 YjbF family lipoprotein [Aeromonas veronii] WP_100654156.1 | 98.60/98.10 | YjbF family lipoprotein |
| 7 | orf4 | 8260..8520 | - | 55.94 | hypothetical protein [Aeromonas sp. DNP9]OEC44539.1 hypothetical protein A9G06_13020 [Aeromonas sp. DNP9] WP_069554774.1 | 98.80/97.70 | hypothetical protein |
| 8 | wzc | 8801..10975 | - | 56.14 | tyrosine-protein kinase [Aeromonas veronii]ATY78184.1 tyrosine-protein kinase [Aeromonas veronii] WP_100654157.1 | 99.40/98.90 | Tyrosine-protein kinase wzc |
| 9 | wzb | 11038..11466 | - | 58.28 | protein-tyrosine-phosphatase [Aeromonas sobria] WP_042018640.1 | 100.00/99.30 | protein-tyrosine-phosphatase |
| 10 | wza | 11702..12817 | - | 58.51 | polysaccharide export protein Wza [Aeromonas sp. DNP9]OEC44542.1 polysaccharide export protein Wza [Aeromonas sp. DNP9] WP_069554777.1 | 100.00/98.70 | polysaccharide export protein |
| 11 | ugd | 13138..14304 | - | 49.27 | nucleotide sugar dehydrogenase [Aeromonas sp. DNP9]OEC44543.1 UDP-glucose 6-dehydrogenase [Aeromonas sp. DNP9] WP_069554778.1 | 99.00/97.20 | UDP-glucose 6-dehydrogenase |
| 12 | GT1 | 14307..15056 | - | 39.71 | MULTISPECIES: glycosyltransferase family 2 protein [Aeromonas] WP_080760772.1 | 99.60/98.80 | glycosyltransferase |
| 13 | GT2 | 15056..15910 | - | 33.33 | MULTISPECIES: hypothetical protein [Aeromonas] WP_080760773.1 | 99.30/98.90 | glycosyltransferase |
| 14 | wbxF | 15989..16504 | - | 37.67 | MULTISPECIES: hypothetical protein [Aeromonas] WP_052248708.1 | 96.40/94.60 | Putative acetyltransferase |
| 15 | GT3 | 16501..17691 | - | 39.46 | hypothetical protein [Pseudoalteromonas lipolytica]KPM84262.1 hypothetical protein AOG27_08140 [Pseudoalteromonas lipolytica] WP_054552517.1 | 72.70/56.20 | Putative glycosyltransferase |
| 16 | orf5 | 17729..18823 | - | 32.42 | hypothetical protein [Pseudoalteromonas rubra]KNC67741.1 hypothetical protein AC626_08865 [Pseudoalteromonas rubra] WP_049864237.1 | 54.90/36.60 | hypothetical protein |
| 17 | GT4 | 18891..19850 | - | 32.81 | hypothetical protein [Aeromonas sp. EERV15] WP_083220970.1 | 61.20/45.70 | Putative glycosyltransferase |
| 18 | vioB | 19888..20457 | - | 40.00 | galactoside O-acetyltransferase [Vibrio parahaemolyticus] WP_025533547.1 | 92.50/79.00 | dTDP-4-amino-4,6-dideoxy-D-glucose acyltransferase |
| 19 | vioA | 20459..21571 | - | 42.14 | aminotransferase [Shewanella sp. W3-18-1]ABM24310.1 DegT/DnrJ/EryC1/StrS aminotransferase [Shewanella sp. W3-18-1] WP_011788811.1 | 94.00/88.30 | dTDP-4-amino-4,6-dideoxy-D-glucose transaminase |
| 20 | wbbK | 21610..23040 | - | 39.97 | lipopolysaccharide biosynthesis protein [Shewanella sp. W3-18-1]ABM24309.1 polysaccharide biosynthesis protein [Shewanella sp. W3-18-1] WP_011788810.1 | 85.10/72.50 | hypothetical protein |
| 21 | rmlA1 | 23046..23921 | - | 40.18 | glucose-1-phosphate thymidylyltransferase [Aeromonas sp. EERV15] WP_068976671.1 | 92.40/85.10 | Glucose-1-phosphate thymidylyltransferase |
| 22 | rmlB1 | 23921..25006 | - | 55.71 | dTDP-glucose 4,6-dehydratase [Aeromonas simiae] WP_042048222.1 | 96.70/95.30 | dTDP-glucose 4,6-dehydratase |
| 23 | GT5 | 25027..26049 | - | 49.66 | MULTISPECIES: exopolysaccharide biosynthesis protein [Aeromonas] WP_043169797.1 | 99.70/99.40 | UDP-N-acetylgalactosamine-undecaprenyl-phosphate N-acetylgalactosaminephosphotransferase |
| 24 | wbgZ | 26822..28807 | - | 53.98 | nucleoside-diphosphate sugar epimerase [Aeromonas veronii]ATY78199.1 nucleoside-diphosphate sugar epimerase [Aeromonas veronii] WP_100654172.1 | 99.80/99.20 | UDP-N-acetyl-alpha-D-glucosamine C6 dehydratase |
| 25 | GT6 | 28804..29682 | - | 54.19 | glycosyl transferase [Aeromonas sp. DNP9]OEC44547.1 glycosyl transferase [Aeromonas sp. DNP9] WP_069554782.1 | 100.00/99.00 | glycosyltransferase |
| 26 | galE | 29829..30785 | - | 53.40 | NAD-dependent dehydratase [Aeromonas veronii]ATY78201.1 NAD-dependent dehydratase [Aeromonas veronii] WP_100654174.1 | 99.70/99.70 | N-acetyl-alpha-D-glucosaminyl-diphospho-ditrans,octacis-undecaprenol 4-epimerase |
| 27 | GT7 | 30785..31588 | - | 41.67 | glycosyl transferase [Aeromonas sp. HZM] WP_050485218.1 | 99.30/98.10 | N-acetylglucosaminyl-diphospho-decaprenol L-rhamnosyltransferase |
| 28 | orf5 | 31660..33366 | - | 32.51 | hypothetical protein PFLuk1_03788 [Pseudomonas fluorescens] KWV68390.1 | 55.50/34.50 | hypothetical protein |
| 29 | GT8 | 33505..35400 | - | 36.45 | glycosyl transferase family 2 [Aeromonas sp. HZM] WP_043155069.1 | 100.00/99.80 | UDP-Glc:alpha-D-GlcNAc-diphosphoundecaprenol beta-1,3-glucosyltransferase |
| 30 | GT9 | 35430..36725 | - | 34.65 | hypothetical protein [Vibrio fluvialis] WP_055453308.1 | 62.30/46.30 | glycosyltransferase |
| 31 | GT10 | 36732..37535 | - | 36.19 | glycosyltransferase family 2 protein [Aeromonas schubertii] WP_082177963.1 | 98.10/94.80 | glycosyltransferase |
| 32 | wzt | 37537..38715 | - | 36.90 | ABC transporter ATP-binding protein [Aeromonas schubertii] WP_082177962.1 | 96.20/91.80 | ABC transporter ATP-binding protein |
| 33 | wzm | 38725..39492 | - | 33.85 | ABC transporter permease [Aeromonas sp. HZM] WP_081844074.1 | 100.00/100.00 | ABC transporter permease |
| 34 | rmlC | 39504..40049 | - | 42.86 | dTDP-4-dehydrorhamnose 3,5-epimerase [Aeromonas sp. HZM] KDV02994.1 | 100.00/100.00 | dTDP-4-dehydrorhamnose 3,5-epimerase |
| 35 | rmlA | 40112..40990 | - | 54.15 | glucose-1-phosphate thymidylyltransferase [Aeromonas caviae] WP_010675334.1 | 99.70/99.30 | Glucose-1-phosphate thymidylyltransferase |
| 36 | rmlD | 41103..41990 | - | 59.91 | NAD(P)-dependent oxidoreductase [Aeromonas veronii] WP_042053006.1 | 99.30/99.00 | dTDP-4-dehydrorhamnose reductase |
| 37 | rmlB | 41990..43081 | - | 56.59 | dTDP-glucose 4,6-dehydratase [Aeromonas veronii] WP_088869799.1 | 99.40/98.30 | dTDP-glucose 4,6-dehydratase |
| 38 | acrB | 43666..46815 | - | 60.57 | multidrug transporter [Aeromonas veronii] WP_058059856.1 | 99.80/99.70 | Multidrug efflux pump subunit AcrB |

***Aeromonas hydrophila*** O24

| **Orf** | **Gene**  **name** | **Location** | **Strand** | **GC**  **Content%** | **Similar protein, strains, Genbank accession number** | **%Identical/**  **%Similar** | **Putative functions** |
| --- | --- | --- | --- | --- | --- | --- | --- |
| 1 | wbpO | 1..1278 | + | 40.61 | UDP-glucose dehydrogenase [Aeromonas hydrophila] AID71077.1 | 93.20/85.20 | UDP-N-acetyl-D-glucosamine 6-dehydrogenase |
| 2 | gne | 1368..2387 | + | 37.45 | UDP-GlcNAc 4-epimerase [Aeromonas hydrophila] AID71076.1 | 88.10/77.40 | UDP-N-acetylglucosamine 4-epimerase |
| 3 | wzx | 2561..3586 | + | 31.79 | Membrane protein involved in the export of O-antigen and teichoic acid [Sphingorhabdus marina DSM 22363] SIO21868.1 | 46.00/27.00 | O antigen flippase |
| 4 | wzy | 3708..4802 | + | 28.77 | hypothetical protein [Vibrio lentus] WP_102362126.1 | 98.20/97.00 | O antigen polymerase |
| 5 | GT1 | 4792..5964 | + | 32.65 | hypothetical protein [Aeromonas veronii] WP_080771371.1 | 100.00/100.00 | glycosyltransferase |
| 6 | GT2 | 5945..7075 | + | 35.37 | hypothetical protein BJP22_13045 [Aeromonas veronii] OKP40062.1 | 100.00/100.00 | glycosyltransferase |
| 7 | wbuX | 7079..8221 | + | 34.91 | N-acetyl sugar amidotransferase [Alishewanella sp. HH-ZS] WP_065956087.1 | 94.10/89.30 | amidotransferase |
| 8 | wbuY | 8218..8835 | + | 39.16 | imidazole glycerol phosphate synthase, glutamine amidotransferase subunit [Alishewanella aestuarii] WP_008608025.1 | 91.70/83.90 | WbuY |
| 9 | wbuZ | 8838..9617 | + | 36.54 | glycosyl amidation-associated protein WbuZ [Pseudomonas cedrina] WP_076950369.1 | 83.10/65.00 | WbuZ |
| 10 | fnlA | 9610..10647 | + | 43.93 | UDP-glucose 4-epimerase [Aeromonas veronii] WP_042052999.1 | 100.00/100.00 | UDP-glucose 4-epimerase |
| 11 | fnlB | 10648..11757 | + | 42.79 | capsular biosynthesis protein [Aeromonas veronii] WP_042053000.1 | 100.00/100.00 | UDP-2-acetamido-2,6-beta-L-arabino-hexul-4-ose reductase |
| 12 | fnlC | 11769..12905 | + | 46.09 | UDP-N-acetyl glucosamine 2-epimerase [Aeromonas veronii] WP_042053034.1 | 100.00/100.00 | UDP-2,3-diacetamido-2,3-dideoxy-D-glucuronate 2-epimerase |
| 13 | GT3 | 12905..14083 | + | 42.24 | glycosyl transferase [Aeromonas veronii] WP_042053001.1 | 100.00/100.00 | glycosyltransferase |
| 14 | galE | 14076..15041 | + | 41.10 | UDP-glucose 4-epimerase [Aeromonas hydrophila] AID71069.1 | 81.70/72.00 | N-acetyl-alpha-D-glucosaminyl-diphospho-ditrans,octacis-undecaprenol 4-epimerase |
| 15 | GT4 | 15044..15598 | + | 44.32 | UDP-N-acetylgalactosaminyltransferase [Aeromonas hydrophila] AID71068.1 | 96.20/94.00 | glycosyltransferase |
| 16 | wbgZ | 15658..17619 | + | 49.80 | epimerase/dehydratase family WbfY-like protein [Aeromonas hydrophila] AID71067.1 | 97.90/95.40 | UDP-N-acetyl-alpha-D-glucosamine C6 dehydratase |
| 17 | rmlB | 17819..18928 | + | 55.50 | dTDP-glucose-4,6-dehydratase [Aeromonas hydrophila] AID70951.1 | 95.10/91.60 | dTDP-glucose 4,6-dehydratase |
| 18 | rmlD | 18928..19815 | + | 60.02 | dTDP-4-dehydrorhamnose reductase [Aeromonas hydrophila] AID71065.1 | 98.00/95.60 | dTDP-4-dehydrorhamnose reductase |
| 19 | rmlA | 19930..20817 | + | 54.39 | glucose-1-phosphate thymidylyltransferase 1 [Aeromonas hydrophila] AID71064.1 | 96.20/94.20 | Glucose-1-phosphate thymidylyltransferase |
| 20 | orf1 | 21320..21087 | + | 50.00 | hypothetical protein [Aeromonas caviae] WP_082189180.1 | 98.50/97.00 | hypothetical protein |
| 21 | rmlC | 22371..22910 | + | 47.78 | dTDP-4-dehydrorhamnose 3,5-epimerase [Aeromonas hydrophila] AID71063.1 | 93.20/88.70 | dTDP-4-dehydrorhamnose 3,5-epimerase |
| 22 | wzb | 22964..24052 | + | 43.53 | hypothetical protein [Aeromonas veronii] WP_005350307.1 | 99.60/97.80 | protein-tyrosine-phosphatase |
| 23 | wza | 24190..26814 | + | 50.93 | polysaccharide export protein [Aeromonas hydrophila] AID70978.1 | 96.50/94.10 | polysaccharide export protein |
| 24 | wzz | 27125..27853 | + | 48.14 | O-antigen chain length determinant protein [Aeromonas hydrophila] AID71062.1 | 93.80/88.40 | O-antigen chain length determinant protein |
| 25 | ymcC | 28279..28950 | + | 42.71 | lipoprotein YmcC [Aeromonas hydrophila] AID70976.1 | 97.30/91.00 | YjbF family lipoprotein |
| 26 | orf2 | 28947..29693 | + | 44.58 | hypothetical protein [Aeromonas veronii] WP_042053017.1 | 100.00/100.00 | hypothetical protein |
| 27 | orf3 | 29693..31765 | + | 44.24 | membrane protein [Aeromonas veronii] WP_042053018.1 | 100.00/100.00 | hypothetical protein |
| 28 | waaL | 31891..33630 | + | 49.20 | ligase [Aeromonas veronii] AMQ43840.1 | 97.40/95.30 | ligase |
| 29 | hns | 34238..34648 | + | 52.31 | DNA-binding protein [Aeromonas veronii] WP_005338054.1 | 99.30/99.30 | DNA-binding protein H-NS |
| 30 | oprM | 35183..36547 | - | 62.34 | transporter [Aeromonas veronii] WP_005359231.1 | 98.50/97.80 | Outer membrane protein OprM |
|  |  |  |  |  |  |  |  |

***Aeromonas hydrophila*** O25

| **Orf** | **Gene**  **name** | **Location** | **Strand** | **GC**  **Content%** | **Similar protein, strains, Genbank accession number** | **%Identical/**  **%Similar** | **Putative functions** |
| --- | --- | --- | --- | --- | --- | --- | --- |
| 1 | waaL | 1..1110 | - | 58.74 | ligase [Aeromonas hydrophila] WP_065476743.1 | 98.90/97.50 | ligase |
| 2 | wzz | 1221..2282 | - | 55.65 | hypothetical protein [Aeromonas hydrophila] WP_044800379.1 | 97.70/96.60 | O-antigen chain length determinant protein |
| 3 | rmlC | 2355..2894 | - | 48.89 | dTDP-4-dehydrorhamnose 3,5-epimerase [Aeromonas hydrophila] WP_081304546.1 | 100.00/100.00 | dTDP-4-dehydrorhamnose 3,5-epimerase |
| 4 | rmlA | 2999..3886 | - | 51.24 | glucose-1-phosphate thymidylyltransferase [Aeromonas hydrophila] WP_065476741.1 | 100.00/99.30 | Glucose-1-phosphate thymidylyltransferase |
| 5 | rmlD | 3999..4886 | - | 61.37 | dTDP-4-dehydrorhamnose reductase [Aeromonas hydrophila] WP_065476739.1 | 99.70/99.30 | dTDP-4-dehydrorhamnose reductase |
| 6 | rmlB | 4886..5974 | - | 57.67 | dTDP-glucose 4,6-dehydratase [Aeromonas hydrophila] WP_043167426.1 | 98.60/97.50 | dTDP-glucose 4,6-dehydratase |
| 7 | wbgZ | 6175..7635 | - | 49.15 | polysaccharide biosynthesis protein [Aeromonas hydrophila] WP_065476736.1 | 100.00/99.60 | UDP-N-acetyl-alpha-D-glucosamine C6 dehydratase |
| 8 | per | 8197..9372 | - | 47.62 | DegT/DnrJ/EryC1/StrS aminotransferase family protein [Aeromonas hydrophila] WP_081304545.1 | 100.00/99.70 | GDP-perosamine synthase |
| 9 | wbnH | 9378..10022 | - | 47.60 | acetyltransferase [Aeromonas hydrophila] WP_081304544.1 | 100.00/99.50 | Putative acetyltransferase |
| 10 | GT1 | 10015..10458 | - | 43.47 | sugar transferase [Aeromonas hydrophila] ANT69978.1 | 100.00/100.00 | glycosyltransferase |
| 11 | GT2 | 10666..11883 | - | 35.47 | glycosyltransferase WbuB [Aeromonas hydrophila] WP_081304543.1 | 100.00/99.30 | glycosyltransferase |
| 12 | wbuX | 11888..13666 | - | 37.21 | glucosamine 6-phosphate synthetase [Aeromonas hydrophila] WP_065480509.1 | 100.00/99.80 | aminotransferase |
| 13 | GT2 | 13751..14812 | - | 34.03 | hypothetical protein [Aeromonas hydrophila] WP_081304542.1 | 99.70/99.70 | glycosyltransferase |
| 14 | wzy | 14900..16345 | - | 33.41 | hypothetical protein BTN33_13330 [Aeromonas veronii] OLF58563.1 | 93.10/88.40 | O antigen polymerase |
| 15 | wzx | 16466..17881 | - | 33.19 | hypothetical protein [Aeromonas hydrophila] WP_081304541.1 | 100.00/99.40 | O antigen flippase |
| 16 | GT3 | 17871..18956 | - | 32.26 | hypothetical protein [Aeromonas hydrophila] WP_081304540.1 | 99.20/98.60 | glycosyltransferase |
| 17 | mnnC | 19059..20138 | - | 42.22 | DegT/DnrJ/EryC1/StrS family aminotransferase [Aeromonas hydrophila] WP_065476732.1 | 100.00/100.00 | mannose-1-phosphate guanylyltransferase |
| 18 | orf1 | 20164..20739 | - | 45.31 | N-acetyltransferase [Aeromonas hydrophila] WP_065476729.1 | 100.00/100.00 | hypothetical protein |
| 19 | gna | 20823..22097 | - | 39.77 | Vi polysaccharide biosynthesis UDP-N-acetylglucosamine C-6 dehydrogenase TviB [Aeromonas hydrophila] WP_065476726.1 | 100.00/100.00 | UDP-GalNAcA synthetase |
| 20 | orf2 | 22113..23159 | - | 42.50 | gfo/Idh/MocA family oxidoreductase [Aeromonas hydrophila] WP_081304539.1 | 100.00/99.70 | hypothetical protein |
| 21 | orf3 | 23494..23688 | - | 46.15 | hypothetical protein [Aeromonas sp. 159] WP_019445673.1 | 76.60/68.10 | hypothetical protein |
| 22 | acrB | 24033..27182 | - | 62.57 | hydrophobe/amphiphile efflux-1 family RND transporter [Aeromonas hydrophila] WP_065476723.1 | 99.30/98.50 | Multidrug efflux pump subunit AcrB |

***Aeromonas hydrophila*** O29

| **Orf** | **Gene**  **name** | **Location** | **Strand** | **GC**  **Content%** | **Similar protein, strains, Genbank accession number** | **%Identical/**  **%Similar** | **Putative functions** |
| --- | --- | --- | --- | --- | --- | --- | --- |
| 1 | rmlA | 1..879 | + | 54.72 | glucose-1-phosphate thymidylyltransferase RfbA [Aeromonas aquatica] WP_073350972.1 | 99.00/98.30 | Glucose-1-phosphate thymidylyltransferase |
| 2 | vioB | 1596..2162 | + | 34.04 | acyltransferase [Escherichia coli] WP_089584058.1 | 84.20/75.00 | dTDP-4-amino-4,6-dideoxy-D-glucose acyltransferase |
| 3 | wvdC | 2174..2872 | + | 33.76 | beta-ketoacyl-ACP reductase [Pseudoalteromonas luteoviolacea] WP_063370149.1 | 73.80/60.70 | reductase |
| 4 | fdtA | 2896..4026 | + | 37.49 | dTDP-4-amino-4,6-dideoxygalactose transaminase [Aeromonas salmonicida] WP_099993903.1 | 92.30/84.00 | dTDP-4-amino-4,6-dideoxygalactose transaminase |
| 5 | GT1 | 4023..5090 | + | 34.46 | hypothetical protein [Shewanella algae] WP_071237815.1 | 81.10/67.90 | glycosyltransferase |
| 6 | wzx | 5244..6548 | + | 36.25 | polysaccharide biosynthesis protein [Shewanella algae] WP_071237814.1 | 83.50/69.40 | O antigen flippase |
| 7 | gnaA | 6648..7919 | + | 37.81 | nucleotide sugar dehydrogenase [Citrobacter sp. NLAE-zl-C269] WP_090049767.1 | 83.50/67.60 | UDP-N-acetyl-D-glucosamine 6-dehydrogenase |
| 8 | GT2 | 7979..9079 | + | 34.97 | hypothetical protein [Citrobacter sp. NLAE-zl-C269] WP_090049769.1 | 76.80/57.90 | glycosyltransferase |
| 9 | wzy | 9143..10222 | + | 29.17 | hypothetical protein [Shewanella sp. POL2] WP_037424696.1 | 50.10/29.30 | O antigen polymerase |
| 10 | GT3 | 10245..11315 | + | 37.82 | glycosyl transferase [Aeromonas australiensis] WP_040094848.1 | 99.40/98.00 | glycosyltransferase |
| 11 | wecA | 11430..12467 | + | 39.02 | undecaprenyl-phosphate alpha-N-acetylglucosaminyl 1-phosphate transferase [Aeromonas australiensis] WP_040094847.1 | 98.60/97.70 | Undecaprenyl-phosphate alpha-N-acetylglucosaminyl 1-phosphate transferase |
| 12 | wzz | 12588..13646 | + | 48.35 | hypothetical protein [Aeromonas australiensis] WP_040094846.1 | 96.30/93.50 | O-antigen chain length determinant protein |
| 13 | waaL | 13750..15480 | + | 49.11 | ligase [Aeromonas veronii] OCQ44529.1 | 91.50/87.30 | ligase |
| 14 | hns | 15953..16363 | + | 53.53 | DNA-binding protein [Aeromonas] WP_005344269.1 | 100.00/100.00 | DNA-binding protein H-NS |
| 15 | oprM | 16900..18264 | - | 62.20 | transporter [Aeromonas veronii] WP_005359231.1 | 98.20/97.60 | Outer membrane protein OprM |

***Aeromonas hydrophila*** O30

| **Orf** | **Gene**  **name** | **Location** | **Strand** | **GC**  **Content%** | **Similar protein, strains, Genbank accession number** | **%Identical/**  **%Similar** | **Putative functions** |
| --- | --- | --- | --- | --- | --- | --- | --- |
| 1 | oprM | 1..1365 | + | 62.05 | transporter [Aeromonas veronii] | 99.10/98.50 | Outer membrane protein OprM |
| 2 | orf1 | 1323..1643 | - | 57.94 | transporter [Aeromonas veronii] WP_075115455.1 | 67.00/46.00 | hypothetical protein |
| 3 | hns | 1903..2313 | - | 53.77 | hypothetical protein [Pseudoglutamicibacter cumminsii] WP_109303974.1 | 100.00/100.00 | DNA-binding protein H-NS |
| 4 | waaL | 2923..4659 | - | 49.68 | DNA-binding protein [Aeromonas] WP_005344269.1 | 97.60/95.90 | ligase |
| 5 | orf2 | 4785..6857 | - | 44.48 | ligase [Aeromonas veronii] WP_042053019.1 | 99.60/99.00 | hypothetical protein |
| 6 | orf3 | 6857..7603 | - | 44.58 | YjbH domain-containing protein [Aeromonas veronii] WP_080771374.1 | 99.60/99.60 | hypothetical protein |
| 7 | ymcC | 7600..8271 | - | 42.11 | hypothetical protein [Aeromonas allosaccharophila] WP_042657814.1 | 99.10/97.30 | YjbF family lipoprotein |
| 8 | orf4 | 8362..8601 | | 50.42 | YjbF family lipoprotein [Aeromonas veronii] WP_042053015.1 | 100.00/100.00 | hypothetical protein |
| 9 | wzz | 8698..9663 | - | 49.07 | hypothetical protein [Aeromonas sp. HMWF015] WP_108613095.1 | 96.70/93.40 | O-antigen chain length determinant protein |
| 10 | wza | 9737..12361 | - | 50.78 | LPS biosynthesis protein [Aeromonas veronii] WP_042053013.1 | 99.00/99.00 | polysaccharide export protein |
| 11 | wzb | 12499..13587 | - | 43.16 | O-antigen chain length determinant protein [Aeromonas veronii] WP_042053011.1 | 95.60/90.30 | protein-tyrosine-phosphatase |
| 12 | rmlD | 14211..15110 | - | 55.56 | protein-tyrosine-phosphatase [Aeromonas veronii] WP_042081901.1 | 80.50/70.20 | dTDP-4-dehydrorhamnose reductase |
| 13 | wecA | 15097..16161 | - | 46.57 | RmlD [Aeromonas hydrophila] AHW40510.1 | 89.20/81.70 | Undecaprenyl-phosphate alpha-N-acetylglucosaminyl 1-phosphate transferase |
| 14 | manB | 16219..17646 | - | 47.20 | undecaprenyl-phosphate alpha-N-acetylglucosaminyl 1-phosphate transferase [Aeromonas australiensis] WP_040094847.1 | 90.60/84.50 | phosphomannomutase |
| 15 | orf4 | 18175..18462 | - | 52.08 | phosphomannomutase [Aeromonas sobria] WP_101347525.1 | 67.00/58.20 | hypothetical protein |
| 16 | orf5 | 18763..18497 | - | 50.56 | hypothetical protein [Escherichia coli] WP_032235255.1 | 67.80/57.80 | hypothetical protein |
| 17 | manC | 18930..20342 | - | 44.73 | hypothetical protein AWN70_25595 [Escherichia coli] KXU60244.1 | 87.00/75.60 | Mannose-1-phosphate guanylyltransferase |
| 18 | GT2 | 20335..21318 | - | 29.17 | mannose-1-phosphate guanylyltransferase/mannose-6-phosphate isomerase [Xenorhabdus stockiae] WP_099124573.1 | 65.20/46.00 | glycosyltransferase |
| 19 | wfaD | 21329..21883 | - | 33.87 | hypothetical protein [Vibrio toranzoniae] WP_060466976.1 | 87.90/75.30 | Putative acetyltransferase |
| 20 | GT3 | 21883..22674 | - | 33.71 | acyltransferase [Vibrio splendidus] WP_081230160.1 | 73.90/55.70 | putative glycosyltransferase |
| 21 | GT4 | 22671..23747 | - | 31.01 | hypothetical protein [Vibrio splendidus] WP_081230161.1 | 74.10/54.00 | glycosyltransferase |
| 22 | wzy | 23772..24929 | - | 30.31 | glycosyltransferase family 4 protein [Vibrio splendidus] WP_081230162.1 | 61.70/41.80 | O antigen polymerase |
| 23 | GT5 | 24916..26034 | - | 31.19 | hypothetical protein [Enterobacter asburiae] WP_087822988.1 | 64.00/41.20 | glycosyltransferase |
| 24 | wzx | 26031..27401 | - | 32.39 | hypothetical protein [Vibrio cyclitrophicus] WP_016788441.1 | 76.50/59.10 | O antigen flippase |
| 25 | tll | 27398..28225 | - | 45.89 | hypothetical protein [Vibrio toranzoniae] WP_060466970.1 | 91.40/85.10 | dTDP-6-deoxy-L-lyxo-4-hexulose reductases |
| 26 | rmlC | 28228..28779 | - | 48.91 | dTDP-glucose 4,6-dehydratase [Aeromonas hydrophila] WP_045527523.1 | 100.00/99.50 | dTDP-4-dehydrorhamnose 3,5-epimerase |
| 27 | rmlA | 28791..29654 | - | 49.65 | dTDP-4-dehydrorhamnose 3,5-epimerase [Aeromonas hydrophila] WP_017410382.1 | 99.00/98.60 | Glucose-1-phosphate thymidylyltransferase |
| 28 | rmlB | 29654..30739 | - | 54.97 | MULTISPECIES: glucose-1-phosphate thymidylyltransferase [Aeromonas] WP_010633428.1 | 97.50/96.40 | dTDP-glucose 4,6-dehydratase |
| 29 | acrB | 31321..34470 | - | 60.51 | dTDP-glucose 4,6-dehydratase [Aeromonas veronii] WP_043818674.1 | 99.80/99.60 | Multidrug efflux pump subunit AcrB |
|  |  |  |  |  | multidrug efflux RND transporter permease subunit [Aeromonas veronii] WP_040065948.1 | | |

***Aeromonas hydrophila*** O33

| **Orf** | **Gene**  **name** | **Location** | **Strand** | **GC**  **Content%** | **Similar protein, strains, Genbank accession number** | **%Identical/**  **%Similar** | **Putative functions** |
| --- | --- | --- | --- | --- | --- | --- | --- |
| 1 | waaL | 1..1731 | - | 61.99 | ligase [Aeromonas hydrophila] WP_050438096.1 | 97.90/96.50 | ligase |
| 2 | orf1 | 1824..3908 | - | 48.20 | YjbH domain-containing protein [Aeromonas hydrophila] WP_101149051.1 | 100.00/100.00 | hypothetical protein |
| 3 | orf2 | 3905..4663 | - | 45.46 | hypothetical protein [Aeromonas hydrophila] WP_080689604.1 | 97.60/96.40 | hypothetical protein |
| 4 | ymcC | 4660..5349 | - | 45.80 | hypothetical protein [Aeromonas hydrophila] WP_011706677.1 | 98.70/98.70 | YjbF family lipoprotein |
| 5 | orf3 | 5400..5648 | - | 55.82 | hypothetical protein [Aeromonas] WP_076361318.1 | 100.00/100.00 | hypothetical protein |
| 6 | wzc | 5796..7970 | - | 54.62 | tyrosine-protein kinase [Aeromonas] WP_076361317.1 | 99.60/98.90 | Tyrosine-protein kinase wzc |
| 7 | wzb | 8033..8461 | - | 59.67 | protein tyrosine phosphatase [Aeromonas sobria] WP_101324075.1 | 99.30/98.60 | protein-tyrosine-phosphatase |
| 8 | wza | 8697..9812 | - | 58.60 | polysaccharide export protein Wza [Aeromonas veronii] WP_100654159.1 | 99.50/98.40 | polysaccharide export protein |
| 9 | ugd | 10133..11299 | - | 49.53 | nucleotide sugar dehydrogenase [Aeromonas veronii] WP_100654161.1 | 99.20/98.70 | UDP-glucose 6-dehydrogenase |
| 10 | wbgU | 11312..12325 | - | 47.34 | KR domain-containing protein [Aeromonas veronii] WP_100654162.1 | 99.10/98.20 | UDP-N-acetylglucosamine 4-epimerase |
| 11 | GT1 | 12337..13455 | - | 33.96 | glycosyltransferase family 4 protein [Aeromonas sp. ZOR0001] WP_081990735.1 | 49.50/34.80 | glycosyltransferase |
| 12 | GT2 | 13452..14528 | - | 33.80 | hypothetical protein [Morganella psychrotolerans] WP_067399513.1 | 59.90/35.00 | glycosyltransferase |
| 13 | orf3 | 14491..15675 | - | 30.97 | hypothetical protein [Proteus mirabilis] WP_049196654.1 | 64.70/44.30 | hypothetical protein |
| 14 | orf4 | 15680..16183 | + | 39.68 | acyltransferase [Proteus mirabilis] WP_080973791.1 | 79.00/70.00 | hypothetical protein |
| 15 | GT3 | 16180..17004 | - | 32.00 | hypothetical protein [Vibrio cyclitrophicus] WP_016792909.1 | 68.50/50.70 | glycosyltransferase |
| 16 | wbxF | 16991..17521 | - | 32.58 | acyltransferase [Shewanella sp. ALD9] WP_101086441.1 | 71.10/56.00 | Putative acetyltransferase |
| 17 | csaB | 17518..18750 | - | 32.85 | hypothetical protein [Vibrio cyclitrophicus] WP_016796070.1 | 64.90/43.70 | hypothetical protein |
| 18 | wecA | 19152..20105 | - | 50.63 | undecaprenyl-phosphate alpha-N-acetylglucosaminyl 1-phosphate transferase [Aeromonas veronii] WP_100654171.1 | 99.70/99.40 | Undecaprenyl-phosphate alpha-N-acetylglucosaminyl 1-phosphate transferase |
| 19 | wbgZ | 20584..22569 | - | 52.57 | polysaccharide biosynthesis protein [Aeromonas hydrophila] WP_101148998.1 | 99.70/99.50 | UDP-N-acetyl-alpha-D-glucosamine C6 dehydratase |
| 20 | GT5 | 22566..23591 | - | 54.00 | glycosyl transferase [Aeromonas hydrophila] WP_101148999.1 | 99.70/99.70 | glycosyltransferase |
| 21 | galE | 23591..24547 | - | 52.77 | NAD-dependent dehydratase [Aeromonas sobria] WP_101347543.1 | 95.60/94.30 | N-acetyl-alpha-D-glucosaminyl-diphospho-ditrans,octacis-undecaprenol 4-epimerase |
| 22 | GT6 | 24547..25356 | - | 46.91 | glycosyl transferase [Aeromonas hydrophila] WP_101149000.1 | 100.00/100.00 | N-acetylglucosaminyl-diphospho-decaprenol L-rhamnosyltransferase |
| 23 | cysC | 25353..25958 | - | 46.04 | adenylyl-sulfate kinase [Aeromonas hydrophila] WP_101149001.1 | 100.00/100.00 | putative adenylyl-sulfate kinase |
| 24 | citT | 25971..27707 | - | 49.28 | SLC13 family permease [Aeromonas hydrophila] WP_101149002.1 | 99.80/99.80 | hypothetical protein |
| 25 | cysN | 27707..29113 | - | 48.61 | sulfate adenylyltransferase subunit CysN [Aeromonas hydrophila] WP_101149003.1 | 100.00/100.00 | Sulfate adenylyltransferase subunit 1 |
| 26 | cysD | 29115..30008 | - | 44.30 | sulfate adenylyltransferase subunit CysD [Aeromonas hydrophila] WP_101149004.1 | 100.00/100.00 | Sulfate adenylyltransferase subunit 2 |
| 27 | fcf1 | 30165..31169 | - | 39.01 | NAD-dependent epimerase [Aeromonas hydrophila] WP_101149053.1 | 100.00/100.00 | dTDP-4-dehydro-6-deoxyglucose reductase |
| 28 | GT7 | 31169..33394 | - | 40.21 | hypothetical protein [Aeromonas hydrophila] WP_101149005.1 | 99.90/99.90 | Putative glycosyltransferase |
| 29 | GT8 | 33397..38853 | - | 38.68 | glycosyl transferase family protein [Aeromonas hydrophila] PKD26094.1 | 98.70/98.40 | Putative glycosyltransferase |
| 30 | wzt | 39004..40389 | - | 38.89 | ABC transporter ATP-binding protein [Aeromonas hydrophila] WP_101149007.1 | 100.00/100.00 | ABC transporter ATP-binding protein |
| 31 | wzm | 40379..41197 | - | 40.17 | ABC transporter permease [Aeromonas hydrophila] WP_101149008.1 | 100.00/100.00 | ABC transporter permease |
| 32 | GT9 | 41277..42230 | - | 35.95 | glycosyltransferase family 2 protein [Aeromonas hydrophila] WP_101149009.1 | 100.00/100.00 | Putative glycosyltransferase |
| 33 | rmlC | 42405..42953 | - | 44.26 | dTDP-4-dehydrorhamnose 3,5-epimerase [Aeromonas hydrophila] WP_101149010.1 | 99.50/99.50 | dTDP-4-dehydrorhamnose 3,5-epimerase |
| 34 | rmlD | 44075..44962 | - | 59.69 | dTDP-4-dehydrorhamnose reductase [Aeromonas hydrophila] WP_101149012.1 | 100.00/100.00 | dTDP-4-dehydrorhamnose reductase |
| 35 | rmlB | 44962..46047 | - | 55.80 | dTDP-glucose 4,6-dehydratase [Aeromonas hydrophila] WP_101149013.1 | 99.70/99.70 | dTDP-glucose 4,6-dehydratase |
| 36 | oprM | 46831..48012 | - | 65.23 | multidrug transporter [Aeromonas hydrophila] WP_049048838.1 | 99.20/98.20 | Outer membrane protein OprM |
| 37 | acrB | 48063..49490 | - | 63.38 | multidrug efflux RND transporter permease subunit [Aeromonas hydrophila] WP_016351371.1 | 100.00/100.00 | Multidrug efflux pump subunit AcrB |

***Aeromonas hydrophila*** O35

| **Orf** | **Gene**  **name** | **Location** | **Strand** | **GC**  **Content%** | **Similar protein, strains, Genbank accession number** | **%Identical/**  **%Similar** | **Putative functions** |
| --- | --- | --- | --- | --- | --- | --- | --- |
| 1 | oprM | 1..1365 | + | 61.76 | transporter [Aeromonas] WP_069527417.1 | 98.70/98.70 | Outer membrane protein OprM |
| 2 | hns | 1902..2312 | - | 52.80 | DNA-binding protein [Aeromonas veronii] WP_005338054.1 | 100.00/100.00 | DNA-binding protein H-NS |
| 3 | waaL | 2868..4523 | - | 49.03 | ligase [Aeromonas veronii] WP_100654834.1 | 99.30/99.00 | ligase |
| 4 | orf1 | 4702..6813 | - | 51.75 | YjbH domain-containing protein [Aeromonas sp. DNP9] WP_069554771.1 | 99.70/99.60 | hypothetical protein |
| 5 | orf2 | 6813..7559 | - | 55.15 | hypothetical protein [Aeromonas veronii] WP_042080341.1 | 98.40/98.00 | hypothetical protein |
| 6 | ymcC | 7556..8200 | - | 50.08 | YjbF family lipoprotein [Aeromonas veronii] WP_100654156.1 | 99.50/99.50 | YjbF family lipoprotein |
| 7 | orf3 | 8258..8518 | - | 57.09 | hypothetical protein [Aeromonas sp. DNP9] WP_069554774.1 | 100.00/100.00 | hypothetical protein |
| 8 | wzc | 8799..10973 | - | 56.41 | tyrosine-protein kinase [Aeromonas veronii] WP_100654157.1 | 99.40/99.40 | Tyrosine-protein kinase wzc |
| 9 | wzb | 11036..11464 | - | 58.97 | protein-tyrosine-phosphatase [Aeromonas sobria] WP_042018640.1 | 100.00/99.30 | protein-tyrosine-phosphatase |
| 10 | wza | 11700..12815 | - | 57.71 | polysaccharide export protein Wza [Aeromonas sp. DNP9] WP_069554777.1 | 99.50/98.40 | polysaccharide export protein |
| 11 | wecA | 13007..14185 | - | 39.95 | glycosyl transferase [Aeromonas veronii] WP_042080348.1 | 90.50/81.10 | Undecaprenyl-phosphate alpha-N-acetylglucosaminyl 1-phosphate transferase |
| 12 | manB | 14201..15619 | - | 46.65 | phosphomannomutase [Aeromonas caviae] KGY67620.1 | 91.50/82.80 | Phosphoglucosamine mutase |
| 13 | manC | 15623..17050 | - | 44.54 | mannose-1-phosphate guanylyltransferase [Shewanella decolorationis S12] ESE41261.1 | 94.20/89.50 | Mannose-1-phosphate guanylyltransferase |
| 14 | GT1 | 17070..18176 | - | 32.79 | WffQ [Shigella dysenteriae] ACD37112.1 | 72.40/51.40 | glycosyltransferase |
| 15 | orf4 | 18176..19369 | - | 31.49 | hypothetical protein [Escherichia coli] WP_001360309.1 | 72.00/52.10 | hypothetical protein |
| 16 | GT2 | 19370..20125 | - | 36.64 | hypothetical protein [Shewanella xiamenensis] WP_069453210.1 | 81.00/66.00 | glycosyltransferase |
| 17 | wffR | 20203..21141 | - | 36.85 | polysaccharide pyruvyl transferase [Vibrio campbellii] WP_045456133.1 | 70.30/50.00 | Putative pyruvyl transferase |
| 18 | orf5 | 21128..22432 | - | 37.24 | hypothetical protein [Vibrio mimicus] WP_000169440.1 | 88.30/81.50 | hypothetical protein |
| 19 | GT3 | 22572..23087 | - | 49.61 | exopolysaccharide biosynthesis protein [Aeromonas caviae] WP_080770111.1 | 100.00/100.00 | glycosyltransferase |
| 20 | GT4 | 23056..23598 | - | 49.36 | exopolysaccharide biosynthesis protein [Aeromonas] WP_043169797.1 | 98.90/98.90 | glycosyltransferase |
| 21 | wbgZ | 24369..26354 | - | 54.08 | nucleoside-diphosphate sugar epimerase [Aeromonas veronii] WP_042080969.1 | 99.80/99.50 | UDP-N-acetyl-alpha-D-glucosamine C6 dehydratase |
| 22 | GT5 | 26351..27376 | - | 53.80 | glycosyl transferase [Aeromonas sp. DNP9] WP_069554782.1 | 99.00/97.60 | glycosyltransferase |
| 23 | galE | 27376..28380 | - | 51.64 | NAD-dependent dehydratase [Aeromonas veronii] WP_100654174.1 | 97.10/96.10 | N-acetyl-alpha-D-glucosaminyl-diphospho-ditrans,octacis-undecaprenol 4-epimerase |
| 24 | GT6 | 28346..29470 | - | 37.42 | glycosyl transferase family 1 [Aeromonas] WP_043169748.1 | 96.80/93.30 | glycosyltransferase |
| 25 | gmm | 29448..29936 | - | 34.36 | GDP-mannose mannosyl hydrolase [Aeromonas] WP_108539984.1 | 100.00/100.00 | GDP-mannose mannosyl hydrolase |
| 26 | orf6 | 29953..30573 | - | 40.58 | acetyltransferase [Aeromonas hydrophila] WP_011706697.1 | 94.10/87.30 | hypothetical protein |
| 27 | GT7 | 30575..32215 | - | 36.93 | glycoside hydrolase [Aeromonas hydrophila] WP_011706698.1 | 95.80/89.40 | glycosyltransferase |
| 28 | GT8 | 32206..34491 | - | 37.23 | glycosytransferase [Aeromonas] WP_043169750.1 | 95.00/88.70 | Putative glycosyltransferase |
| 29 | wzt | 34488..35246 | - | 37.81 | polysaccharide/polyol phosphate ABC transporter ATP-binding protein [Aeromonas hydrophila] WP_011706700.1 | 98.00/96.80 | ABC transporter ATP-binding protein |
| 30 | wzm | 35243..36028 | - | 38.30 | ABC transporter permease [Aeromonas hydrophila] WP_011706701.1 | 97.70/96.90 | ABC transporter permease |
| 31 | per | 36092..37180 | - | 37.65 | DegT/DnrJ/EryC1/StrS aminotransferase family protein [Aeromonas] WP_080760780.1 | 96.40/93.40 | GDP-perosamine synthase |
| 32 | gmd | 37183..38304 | - | 42.60 | GDP-mannose 4,6-dehydratase [Aeromonas hydrophila] WP_011706703.1 | 99.50/98.10 | GDP-mannose 4,6-dehydratase |
| 33 | manB | 38319..39728 | - | 39.72 | phosphomannomutase [Aeromonas] WP_080760782.1 | 96.30/94.50 | Phosphomannomutase/phosphoglucomutase |
| 34 | manC | 39709..41106 | - | 39.13 | mannose-1-phosphate guanylyltransferase/mannose-6-phosphate isomerase [Aeromonas] WP_080760783.1 | 96.80/92.50 | Mannose-1-phosphate guanylyltransferase |
| 35 | rmlC | 41145..41690 | - | 38.83 | dTDP-4-dehydrorhamnose 3,5-epimerase [Aeromonas hydrophila] WP_011706706.1 | 92.30/80.10 | dTDP-4-dehydrorhamnose 3,5-epimerase |
| 36 | rmlA | 41753..42631 | - | 51.31 | glucose-1-phosphate thymidylyltransferase [Aeromonas veronii] WP_021231189.1 | 99.00/95.20 | Glucose-1-phosphate thymidylyltransferase |
| 37 | rmlD | 42743..43630 | - | 60.47 | dTDP-4-dehydrorhamnose reductase [Aeromonas veronii] AMQ43866.1 | 98.30/96.60 | dTDP-4-dehydrorhamnose reductase |
| 38 | rmlB | 43630..44736 | - | 57.09 | dTDP-glucose 4,6-dehydratase [Aeromonas sp. ZOR0001] WP_047439253.1 | 98.60/96.50 | dTDP-glucose 4,6-dehydratase |
| 39 | acrB | 45316..48465 | - | 60.73 | multidrug efflux RND transporter permease subunit [Aeromonas veronii] WP_042084574.1 | 99.90/99.50 | Multidrug efflux pump subunit AcrB |

***Aeromonas hydrophila*** O44

| **Orf** | **Gene**  **name** | **Location** | **Strand** | **GC**  **Content%** | **Similar protein, strains, Genbank accession number** | **%Identical/**  **%Similar** | **Putative functions** |
| --- | --- | --- | --- | --- | --- | --- | --- |
| 1 | acrB | 1..1812 | + | 63.02 | multidrug efflux RND transporter permease subunit [Aeromonas hydrophila] WP_041217358.1 | 100.00/100.00 | Multidrug efflux pump subunit AcrB |
| 2 | orf1 | 1793..3151 | + | 63.21 | multidrug efflux RND transporter permease subunit [Aeromonas hydrophila] WP_044800392.1 | 100.00/100.00 | hypothetical protein |
| 3 | oprM | 3144..4553 | + | 65.60 | multidrug transporter [Aeromonas hydrophila] WP_039213772.1 | 99.60/98.90 | Outer membrane protein OprM |
| 4 | orf2 | 5062..5193 | + | 47.73 | hypothetical protein - | | hypothetical protein |
| 5 | rmlB | 5253..5513 | + | 49.43 | dTDP-glucose 4,6-dehydratase [Aeromonas hydrophila] WP_049084120.1 | 83.00/77.00 | dTDP-glucose 4,6-dehydratase |
| 6 | wzy | 6339..7547 | + | 30.60 | MutG family lantibiotic protection ATP binding cassette transporter permease subunit [Aeromonas bestiarum] AID70973.1 | 84.40/70.30 | O antigen polymerase |
| 7 | wzx | 7537..8706 | + | 28.29 | polysaccharide biosynthesis protein [Aeromonas bestiarum] AID70972.1 | 83.20/70.30 | O antigen flippase |
| 8 | GT1 | 8708..9493 | + | 30.28 | glycosyl transferase, group 2 family protein [Aeromonas bestiarum] AID70971.1 | 88.10/77.80 | glycosyltransferase |
| 9 | werO | 9478..10059 | + | 32.99 | acetyltransferase [Aeromonas bestiarum] AID70970.1 | 94.80/83.90 | acetyltransferase |
| 10 | GT2 | 10044..11018 | + | 31.59 | glycosyl transferase, group 1 family protein [Aeromonas bestiarum] AID70969.1 | 81.80/62.70 | glycosyltransferase |
| 11 | GT3 | 12237..13100 | + | 31.37 | glycosyl transferase, group 2 family protein [Aeromonas bestiarum] AID70968.1 | 77.70/65.20 | glycosyltransferase |
| 12 | GT4 | 13100..14089 | + | 32.53 | glycosyl transferase, group 1 family protein [Aeromonas bestiarum] AID70967.1 | 90.00/79.60 | glycosyltransferase |
| 13 | gmd | 14103..15215 | + | 50.58 | GDP-D-mannose dehydratase [Aeromonas bestiarum] AID70966.1 | 96.20/93.00 | GDP-mannose 4,6-dehydratase |
| 14 | fcl | 15219..16196 | + | 54.50 | GDP-fucose synthetase [Aeromonas allosaccharophila] WP_042657821.1 | 95.70/93.50 | GDP-L-fucose synthase |
| 15 | gmm | 16193..16672 | + | 51.46 | GDP-mannose mannosyl hydrolase [Aeromonas caviae] KGY67622.1 | 99.00/91.10 | GDP-mannose mannosyl hydrolase |
| 16 | manC | 16677..18083 | + | 53.87 | mannose-1-phosphate guanyltransferase [Aeromonas caviae] KGY67621.1 | 97.00/94.70 | Mannose-1-phosphate guanylyltransferase 1 |
| 17 | manB | 18114..19532 | + | 47.08 | phosphomannomutase [Aeromonas allosaccharophila] WP_042657818.1 | 91.40/84.60 | Phosphoglucosamine mutase |
| 18 | GT5 | 19700..20440 | + | 34.14 | glycosyl transferase [Vibrio cholerae] WP_002030636.1 | 87.80/76.40 | glycosyltransferase |
| 19 | galE | 20437..21396 | + | 41.04 | UDP-glucose 4-epimerase [Vibrio harveyi] WP_050902742.1 | 83.90/75.60 | N-acetyl-alpha-D-glucosaminyl-diphospho-ditrans,octacis-undecaprenol 4-epimerase |
| 20 | GT6 | 21393..21947 | + | 45.59 | sugar transferase [Aeromonas sp. SCS5] WP_071911975.1 | 95.70/92.40 | glycosyltransferase |
| 21 | wbgZ | 22007..23968 | + | 48.98 | polysaccharide biosynthesis protein [Aeromonas sp. YN13HZO-058] WP_075113086.1 | 98.80/97.90 | UDP-N-acetyl-alpha-D-glucosamine C6 dehydratase |
| 22 | rmlB | 24167..25255 | + | 57.85 | dTDP-glucose 4,6-dehydratase [Aeromonas hydrophila] WP_043167426.1 | 98.30/97.50 | dTDP-glucose 4,6-dehydratase |
| 23 | rmlD | 25255..26142 | + | 60.36 | dTDP-4-dehydrorhamnose reductase [Shigella boydii] WP_096106376.1 | 98.60/98.60 | dTDP-4-dehydrorhamnose reductase |
| 24 | rmlA | 26254..27141 | + | 51.80 | glucose-1-phosphate thymidylyltransferase [Aeromonas hydrophila] WP_044800381.1 | 99.70/99.00 | Glucose-1-phosphate thymidylyltransferase 1 |
| 25 | rmlC | 27246..27785 | + | 47.59 | dTDP-4-dehydrorhamnose 3,5-epimerase [Aeromonas hydrophila] AID71063.1 | 100.00/100.00 | dTDP-4-dehydrorhamnose 3,5-epimerase |
| 26 | wzz | 27858..28919 | + | 55.37 | hypothetical protein [Aeromonas hydrophila] WP_044800379.1 | 99.70/99.40 | O-antigen chain length determinant protein |
| 27 | waaL | 29014..30732 | + | 62.65 | lipid A core - O-antigen ligase [Aeromonas hydrophila] AID71061.1 | 98.30/97.40 | ligase |
