# Supplementary material for: Identifying genetic diversity of O antigens in Aeromonas hydrophila for molecular serotype detection
Source: PLoS One. 2018 Sep 5;13(9):e0203445. doi: 10.1371/journal.pone.0203445 (PMC6124807; doi:10.1371/journal.pone.0203445)
Supplement: S6 Table — (DOC) [file pone.0203445.s006.doc]

**S6 Table. The homologous groups of glycosyltransferases in *Aeromonas hydrophila*** OGC

| **Homologous Groups** | **Glycosyltransferases** |
| --- | --- |
| HG1 | GT4_O10 GT6_O23 GT6_O35 wbpI_O7 GT5_O13 |
| HG2 | wecA_O10 wecA_O34 GT4_O16 GT4_O35 |
| HG3 | GT4_O13 wbxW_O7 GT7_O23 GT5_O33 |
| HG4 | wecA_O7 wecA_O13 wecA_O33 |
| HG5 | wecA_O9 wecA_O29 wecA_O30 |
| HG6 | GT1_O10 GT1_O35 |
| HG7 | GT5_O10 GT7_O35 |
| HG8 | GT1_O9 GT5_O44 |
| HG9 | GT3_O13 wbxX_O7 |
| HG10 | GT5_O23 GT5_O35 |
| HG11 | GT1_O23 GT1_O7 |
| HG12 | GT4_O24 GT6_O44 |
| HG13 | GT3_O33 GT7_O13 |
| HG14 | GT6_O33 GT7_O33 |
| HG15 | GT4_O44 wbxE_O34 |
