# Supplementary figures and images for: Identifying genetic diversity of O antigens in Aeromonas hydrophila for molecular serotype detection
Source: PLoS One. 2018 Sep 5;13(9):e0203445. doi: 10.1371/journal.pone.0203445 (PMC6124807; doi:10.1371/journal.pone.0203445)

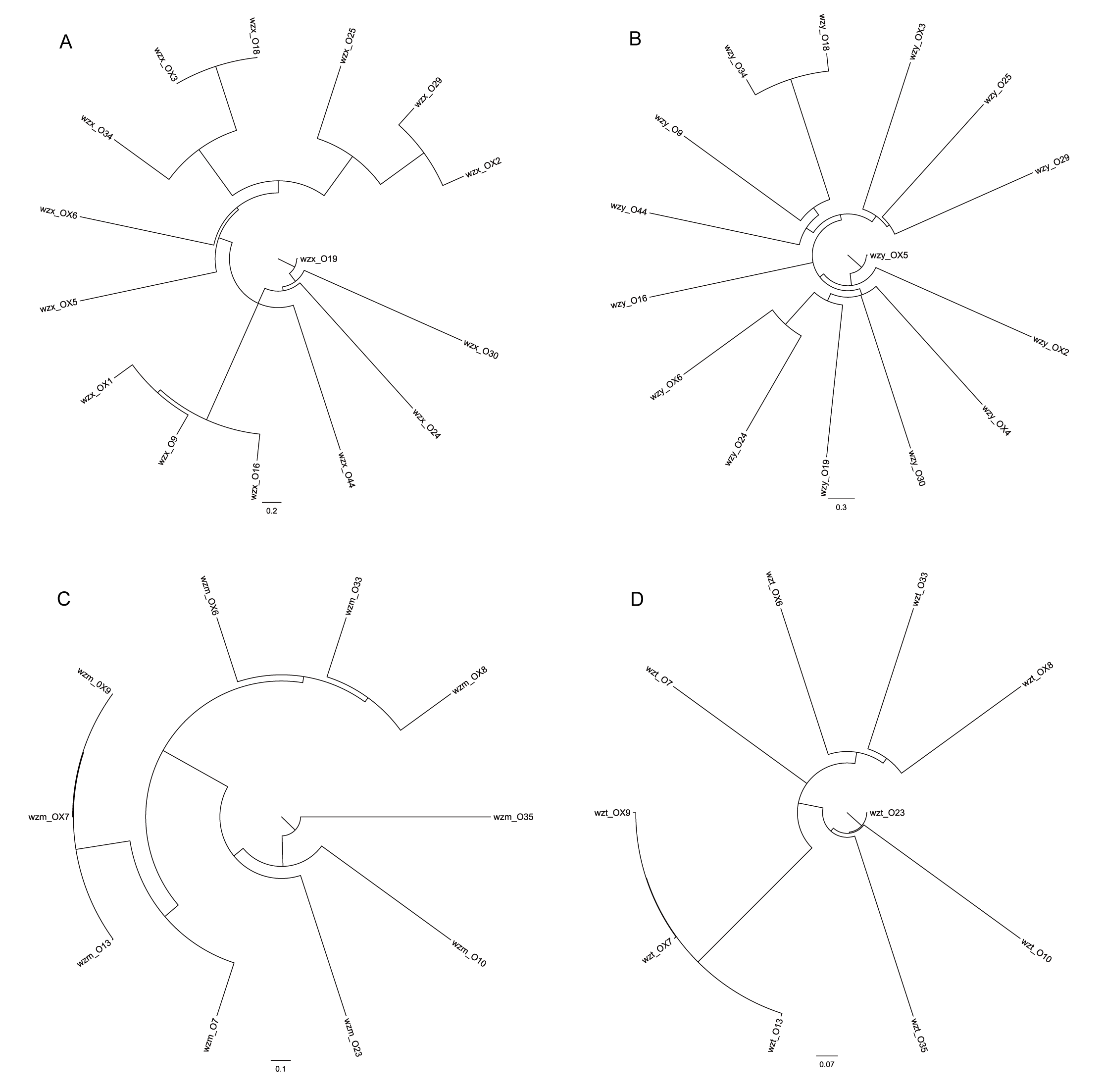

Supplement: S1 Fig — The wzx (A), wzy (B), wzm (C), and wzt (D) trees were constructed using wzx, wzy, wzm, and wzt gene sequences. The sequences were aligned using MUSCLE (v3.8), and the trees were constructed using phyML (v3.0). (TIF) [file pone.0203445.s007.tif]
